# Nasal Suctioning Therapy Among Infants With Bronchiolitis Discharged Home From the Emergency Department: A Randomized Clinical Trial

**DOI:** 10.1001/jamanetworkopen.2023.37810

**Published:** 2023-10-19

**Authors:** Suzanne Schuh, Allan L. Coates, Judy Sweeney, Maggie Rumantir, Mohamed Eltorki, Waleed Alqurashi, Amy C. Plint, Roger Zemek, Naveen Poonai, Patricia C. Parkin, Diane Soares, Rahim Moineddin, Yaron Finkelstein

**Affiliations:** 1 Division of Pediatric Emergency Medicine, The Hospital for Sick Children, University of Toronto, Toronto, Ontario, Canada; 2 Research Institute, The Hospital for Sick Children, University of Toronto, Toronto, Ontario, Canada; 3 Division of Pediatric Emergency Medicine, McMaster Children’s Hospital, Hamilton Health Sciences, Hamilton, Ontario, Canada; 4 Faculty of Health Sciences, McMaster University, Hamilton, Ontario, Canada; 5 Department of Pediatrics and Emergency Medicine, Children’s Hospital of Eastern Ontario, University of Ottawa, Ottawa, Ontario, Canada; 6 CHEO Research Institute, Ottawa, Ontario, Canada; 7 Department of Paediatrics, Schulich School of Medicine & Dentistry, Western University, London, Ontario, Canada; 8 Department of Epidemiology & Biostatistics, Schulich School of Medicine & Dentistry, Western University, London, Ontario, Canada; 9 Children’s Health Research Institute, University of Western Ontario, London, Ontario, Canada; 10 Division of Pediatric Medicine, The Hospital for Sick Children, University of Toronto, Toronto, Ontario, Canada; 11 Department of Respiratory Therapy, The Hospital for Sick Children, University of Toronto, Toronto, Ontario, Canada; 12 Dalla Lana School of Public Health, University of Toronto, Toronto, Ontario, Canada; 13 Division of Clinical Pharmacology and Toxicology, The Hospital for Sick Children, University of Toronto, Toronto, Ontario, Canada

## Abstract

**Importance:**

Although nasal suctioning is the most frequently used supportive management for bronchiolitis, its benefit remains unknown.

**Objective:**

To evaluate the effectiveness of enhanced vs minimal nasal suctioning in treating infants with bronchiolitis after discharge from the emergency department (ED).

**Design, Setting, and Participants:**

This single-blind, parallel-group, randomized clinical trial was conducted from March 6, 2020, to December 15, 2022, at 4 tertiary-care Canadian pediatric EDs. Participants included otherwise healthy infants aged 1 to 11 months with a diagnosis of bronchiolitis who were discharged home from the ED.

**Interventions:**

Participants were randomized to minimal suctioning via bulb or enhanced suctioning via a battery-operated device before feeding for 72 hours.

**Main Outcomes and Measures:**

The primary outcome was additional resource use, a composite of unscheduled revisits for bronchiolitis or use of additional suctioning devices for feeding and/or breathing concerns. Secondary outcomes included health care utilization, feeding and sleeping adequacy, and satisfaction.

**Results:**

Of 884 screened patients, 352 were excluded for criteria, 79 declined participation, 81 were otherwise excluded, 372 were randomized (185 to the minimal suction group and 187 to the enhanced suction group), and 367 (median [IQR] age, 4 [2-6] months; 221 boys [60.2%]) completed the trial (184 in the minimal suction and 183 in the enhanced suction group). Additional resource use occurred for 68 of 184 minimal suction participants (37.0%) vs 48 of 183 enhanced suction participants (26.2%) (absolute risk difference, 0.11; 95% CI, 0.01 to 0.20; *P* = .03). Unscheduled revisits occurred for 47 of 184 minimal suction participants (25.5%) vs 40 of 183 enhanced suction participants (21.9%) (absolute risk difference, 0.04; 95% CI, −0.05 to 0.12; *P* = .46). A total of 33 of 184 parents in the minimal suction group (17.9%) used additional suctioning devices vs 11 of 183 parents in the enhanced suction group (6.0%) (absolute risk difference, 0.12; 95% CI, 0.05 to 0.19; *P* < .001). No significant between-group differences were observed for all bronchiolitis revisits (absolute risk difference, 0.07; 95% CI, −0.02 to 0.16; *P* = .15), ED revisits (absolute risk difference, 0.04; 95% CI, −0.03 to 0.12; *P* = .30), parental care satisfaction (absolute risk difference, −0.02; 95% CI, −0.10 to 0.06; *P* = .70), and changes from baseline to 72 hours in normal feeding (difference in differences, 0.03; 95% CI, −0.10 to 0.17; *P* = .62), normal sleeping (difference in differences, 0.05; 95% CI, −0.08 to 0.18; *P* = .47), or normal parental sleeping (difference in differences, 0.10; 95% CI, −0.02 to 0.23; *P* = .09). Parents in the minimal suction group were less satisfied with the assigned device (62 of 184 [33.7%]) than parents in the enhanced suction group (145 of 183 [79.2%]) (risk difference, 0.45; 95% CI, 0.36 to 0.54; *P* < .001).

**Conclusions and Relevance:**

Compared with minimal suctioning, enhanced suctioning after ED discharge with bronchiolitis did not alter the disease course because there were no group differences in revisits or feeding and sleeping adequacy. Minimal suctioning resulted in higher use of nonassigned suctioning devices and lower parental satisfaction with the assigned device.

**Trial Registration:**

ClinicalTrials.gov Identifier: NCT03361371

## Key Points

**Question:**

What is the effectiveness of enhanced nasal suctioning compared with minimal suctioning in infants with bronchiolitis discharged home from pediatric emergency departments?

**Findings:**

In this randomized clinical trial of 367 infants, minimal bulb suctioning resulted in significantly higher additional resource use compared with enhanced battery-operated suctioning at 72 hours. There was a nonsignificant difference in unscheduled revisits for bronchiolitis, but minimal suctioning yielded significantly higher use of nonassigned suctioning devices for perceived breathing or feeding difficulties than enhanced suctioning.

**Meaning:**

Enhanced suctioning after emergency department discharge with bronchiolitis did not alter the disease course compared with minimal suctioning.

## Teaser

This randomized clinical trial evaluates the effectiveness of enhanced vs minimal nasal suctioning in infants with bronchiolitis discharged home from the emergency department (ED).

## Introduction

Bronchiolitis is the leading cause of infant hospitalizations,^1,2,3^ with substantial associated use of health care resources.^4,5^ Because effective therapies remain elusive, bronchiolitis guidelines advocate for the use of supportive measures only.^6,7,8,9,10,11^ This paucity of effective evidence-based therapies has led to substantial practice variation, including unwarranted treatments.^12,13,14,15,16,17,18^ Therefore, it is important to determine which aspects of supportive care are effective.

Infants are obligate nose breathers,^19^ and nasal congestion in bronchiolitis contributes to respiratory distress, altered sleep cycle, and feeding difficulties.^19,20^ Poor feeding represents a major reason for bronchiolitis hospitalizations.^19^ Therefore, many physicians and parents use measures to relieve nasal congestion.^21,22^

However, there is little information about the benefit of suctioning in bronchiolitis. Bronchiolitis guidelines highlight this limitation and call for evidence to support this practice.^6,7,10,11^ Previous studies^23,24^ of suctioning have largely addressed the treatment of upper respiratory infections; the research of suctioning in bronchiolitis is limited to a nonrandomized inpatient study^25^ and one single-center randomized study,^26^ with suboptimal power and lack of a reference standard. The benefit of suctioning in bronchiolitis, therefore, remains unclear.

To address this knowledge gap, we designed the Suctioning of Nose Therapy in Bronchiolitis Trial (SNOT) to compare the effectiveness of enhanced nasal suctioning via a pretested battery-operated device with that of minimal suctioning via a pretested bulb in infants with bronchiolitis discharged from pediatric emergency departments (EDs). We hypothesized that the infants suctioned via the enhanced method would experience a lower probability of additional resource use within 72 hours after discharge than those receiving minimal suctioning.

## Methods

### Design

This was a multicenter, single-blind, parallel-group, randomized clinical trial comparing the effect of minimal suctioning via a bulb (Life Brand Nasal Aspirator) vs enhanced suctioning via a battery-operated device (Zo-Li, Inc) in infants with bronchiolitis at 4 Canadian tertiary-care Pediatric Emergency Research Canada Network EDs.^27^ Written informed consent was obtained from all caregivers. The research ethics boards at all institutions approved the trial. This study follows the Consolidated Standards of Reporting Trials (CONSORT) reporting guideline for randomized studies.

### Participants

Infants aged 4 weeks (after expected delivery date)^28^ to 11 months were eligible if they had received a diagnosis of bronchiolitis by the treating ED physician and were scheduled for discharge home. Bronchiolitis was defined as the first episode of upper respiratory infection with nasal congestion and respiratory distress.^7^ Infants who received a diagnosis of bronchiolitis more than 3 weeks before the index ED visit were excluded,^29^ as were those with known congenital heart or chronic respiratory disease, severe gastroesophageal reflux, neuromuscular or neurologic disease, immunodeficiency, coagulopathies, nasal or upper airway or oral or gastrointestinal anomalies, and gastric or gastrojejunal tube feeding supplementation. We also excluded children suctioned by any battery-operated devices before arrival (because of concern about continued use of these devices during the study), the families without email or telephone contact, and those unable to communicate in English.

### Device-Finding Prestudy

In preliminary work, we assessed several suctioning devices. The Zo-Li device generated sustained high negative pressure of 126 mm Hg during the 10-second suctioning interval, which is comparable to the accepted Canada-wide wall suction standard.^30^ Suction pressure of the mouth-to-nose suction devices (eg, Hydrasense, Naspira, and Nosefrida) were operator dependent. A previous study^26^ suggested that the benefit of the mouth-to-nose devices may be similar to that for a bulb. The Life Brand Nasal Aspirator bulb had a short time of less than 1 second available for bulb release, with nonsustained aspiration pressures. Therefore, we selected the bulb as the comparator because it could reasonably be expected to provide minimal suctioning effect.

To assess the benefit of suctioning, we considered a control group receiving no suctioning. To that effect, the original protocol comparing enhanced suction with usual care assumed that usual care would largely consist of no suction or bulb use. However, after enrolling 35 infants (December 26, 2018, to February 7, 2019), we discovered that virtually all parents already used suctioning and that almost no parents continued suctioning beyond 3 days. We, therefore, anticipated that no suctioning would make enrollment difficult and might be considered unethical and, thus, selected standardized minimal suctioning via a bulb as the control group. Because of the strong preference for suctioning found during this initial phase, we anticipated that some parents may use unassigned devices for perceived lack of breathing or feeding improvement, which would also represent additional resource use. We, therefore, incorporated unassigned device use with unscheduled revisits into the revised composite primary outcome and changed the intervention length from 7 days to 72 hours. This change was research ethics board–approved and registered on ClinicalTrials.gov. Please see Supplement 1 for the original and amended treatment protocols with the statistical analysis outline. Enrollment of the first 35 participants constituted a pilot phase, and their data were not included in the analysis.

### Procedures

Trained research assistants (none of whom was a coauthor of this study) prescreened potentially eligible patients and confirmed the diagnosis of bronchiolitis with the treating physician. Once the planned discharge home had been communicated and permission to be approached for research obtained, the assistants confirmed eligibility, obtained consent, and collected baseline characteristics. The lack of knowledge about potential differences in benefit between the study devices was emphasized to the families.

The assistants provided the families with the assigned suctioning devices and instructed them in their proper use, in person plus via a prerecorded video. The parents were instructed to suction the nose before each feeding for 72 hours and to avoid using nonassigned devices. The 72-hour intervention period was chosen because most infants with bronchiolitis experience unscheduled revisits within this time.^5^ All participants were given standardized bronchiolitis discharge instructions on the expected symptom duration^31^ and when to seek further care.

The research assistants recorded the vital signs measured by the ED nurses before discharge. A follow-up questionnaire was sent to the caregiver’s email address via secure SickKids REDCap database at 72 hours and was reviewed by an intervention-blinded study manager (J.S. or M.R.) (eAppendix 1 in Supplement 2). The families choosing telephone follow-up were contacted by a research assistant trained in interviewing techniques and blinded to the intervention assignment.

### Intervention

During our prestudy evaluation, we found that the battery-operated device generated sustained, operator-independent high negative pressures, similar to those obtained in the hospital setting, as demonstrated on 10 separate suctioning attempts. In contrast, the pressure generated by the nose-to-mouth devices is highly operator-dependent and approaches 0 by 3 seconds. Therefore, we selected the battery-operated device as the intervention.

### Outcomes

The primary outcome was a composite of additional resource use, defined as (1) any unscheduled family-initiated^32^ or health care practitioner–initiated bronchiolitis-related visit to any medical facility within 72 hours after ED discharge,^5,33,34^ except the visits occurring only because of ED recommendation and without parental concern about bronchiolitis or (2) use of any unassigned suctioning devices, with stated parental concern about the assigned device not helping with feeding or breathing. Both components highlight concern about ongoing symptoms.^35^

Secondary outcomes measured at baseline and 72 hours included parent-reported child feeding and sleep^6^ and caregiver sleep adequacy (eAppendix 2 in Supplement 2). At 72 hours, parents reported any revisits for bronchiolitis, ED revisits, and satisfaction with care for their child’s illness. As an exploratory outcome, we also studied parental satisfaction with the assigned device (eAppendix 3 in Supplement 2).

We tracked the expected bronchiolitis-related adverse events, which included respiratory distress, fever, vomiting, hospitalization after discharge, medical or ED revisits, intravenous or nasogastric hydration, oxygen, postsuctioning crying, and nasal irritation or bleeding without medical intervention. Serious adverse events consisted of admission to an intensive care unit within 72 hours.

### Sample Size

The sample size calculation was based on the assessment of the between-group difference in the primary outcome by the Fisher exact test. According to a published study^34^ and during the pilot phase, the probability of a revisit within 72 hours was estimated at 35%. Study investigators considered an absolute between-group difference in the primary outcome of 15 percentage points as clinically meaningful, representing a number-needed-to-treat of 7. In the Cochrane review of asthma therapies,^36^ a number-needed-to-treat of a comparable magnitude led to a change in national practice recommendations. Because bronchiolitis-related revisits are highly prevalent,^37^ this difference would also have an economic impact. Aiming at a 2-sided significance level of 5%, power of 80%, absolute effect estimate of 15%, and assuming a 10% rate of missing primary outcome for any reason, we planned to randomize 360 participants.

### Randomization

Using random number-generating software,^38^ a third-party treatment allocator produced and securely guarded master randomization tables, stratified by site and age (<6 months vs ≥6 months).^19^ Permuted block randomization with block sizes of 4 and 6 with a 1:1 group allocation ratio was used. The randomization sequence was restricted to the third-party service until the study database was locked. Upon receiving the email indicating the study number and group assignment, the research assistant obtained the appropriate study kit containing standardized bronchiolitis discharge instructions and either the battery-operated device or the bulb device with instructions for device use and entered the study number into a logbook.

### Blinding

Although the caregivers could not be blinded to study intervention, they were blinded to the study hypothesis. The investigators, study managers (J.S. and M.R.), ED staff, research assistants performing telephone follow-ups, and the analyst were blinded. To blind the ED staff, the parents were instructed not to reveal the assigned device to the ED team. To blind the analyst, the assigned allocation in the database was labeled by an unidentified group 1 or group 2 designation.

### Statistical Analysis

All analyses were specified a priori, and no interim analyses were conducted. Baseline characteristics were described using medians and IQRs for continuous variables and frequency and percentages for categorical variables.

All analyses followed the intention-to-treat principle. The between-group difference in primary outcome was assessed using a 2-sided Fisher exact test. The estimated effect was reported as a relative risk difference with corresponding 95% CIs and *P* values. The Fisher exact test was also used for the examination of each of the 2 primary outcome components.

For secondary analyses, the between-group differences in secondary outcomes were similarly assessed using a Fisher exact test and reported as relative risk differences with 95% CIs and *P* values. For both primary and secondary outcomes, we conducted adjusted analyses using generalized linear mixed modeling to control for randomization stratification by age group and site, where the site was treated as a random effect. When the random effect model did not converge owing to lack of site variability, the least square logistic regression was used. Similar methods were used in the per-protocol analyses.

Because adverse events were uncommon, these were reported in a descriptive way. Overall significance for all analyses was set at *P* < .05. Statistical analysis was conducted using SAS statistical software version 9.4 (SAS Institute).

## Results

Of 884 screened patients, 352 were excluded for criteria, 79 declined participation, and 81 were otherwise excluded. Between March 6, 2020, and December 15, 2022, 372 infants were randomized (185 to the minimal suction group and 187 to the enhanced suction group). A total of 184 infants in the enhanced suction group and 183 in the minimal suction group (367 total) completed the follow-up for the primary outcome (Figure). The median (IQR) age of the participants was 4 (2-6) months; there were 221 boys (60.2%). The trial groups had similar baseline characteristics (Table 1).

**Figure.**
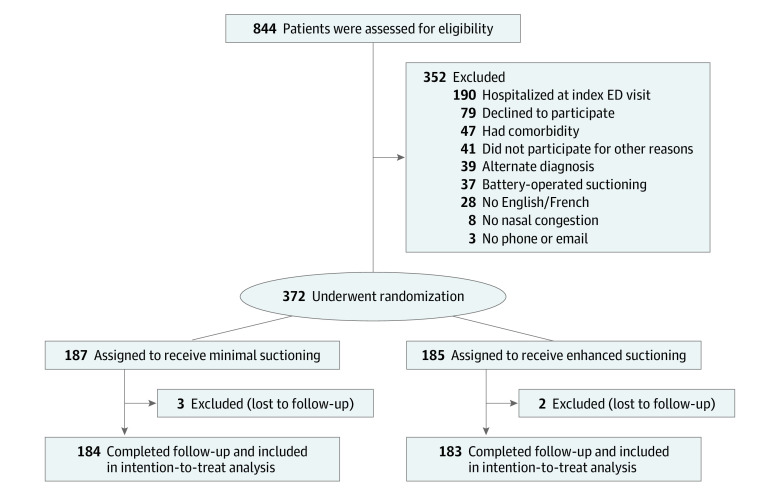
Enrollment and Randomization in the Trial ED indicates emergency department.

**Table 1.** Characteristics of the Enrolled Participants at Randomization

Characteristic	Participants, No. (%) (N = 367)	
	Minimal suction (n = 184)	Enhanced suction (n = 183)
Age, median (IQR), mo	4 (2-6)	4 (2-6)
Age <6 mo	133 (72.3)	134 (73.2)
Sex		
Male	109 (59.2)	112 (61.2)
Female	75 (40.8)	71 (38.8)
Prematurity <37 wk	11 (6.0)	21 (11.5)
Eczema history, No./total No. (%)^a^	33/178 (18.5)	28/179 (15.6)
Atopy in parents or siblings, No./total No. (%)^b^	99/181 (55.0)	91/181 (50.3)
Prior ED visits for this episode	38 (20.6)	38 (20.8)
Duration of respiratory distress, median (IQR), h	24 (5-64)	24 (5-48)
Treatment before arrival		
Oral corticosteroids	12 (6.5)	5 (2.7)
Inhaled albuterol	21 (11.4)	18 (9.8)
Inhaled corticosteroids	7 (3.8)	5 (2.7)
Mouth-to-nose suction	113 (61.4)	105 (57.1)
Bulb suction	40 (21.7)	54 (29.5)
Feeding before arrival, % of normal		
>80	52 (28.3)	56 (30.6)
50-80	97 (52.7)	94 (51.4)
<50	35 (19.0)	33 (18.0)
Sleeping before arrival		
Very much less than normal	37 (20.1)	39 (21.3)
Less than normal	87 (47.3)	72 (39.3)
About normal	31 (16.8)	40 (21.9)
More than normal	23 (12.5)	20 (10.9)
Very much more than normal	6 (3.3)	12 (6.6)
Parental sleep before arrival		
Very much less than normal	101 (54.9)	87 (47.5)
Less than normal	54 (29.3)	57 (31.1)
About normal	27 (14.7)	28 (15.3)
More than normal	1 (0.5)	5 (2.7)
Very much more than normal	1 (0.5)	6 (3.3)
Disease severity		
Respiratory rate, median (IQR), breaths/min	48 (40-54)	46 (40-52)
Heart rate, median (IQR), beats/min	148 (140-160)	148 (136-158)
Oxygen saturation, median (IQR), %	98 (96-99)	98 (96-99)
Temperature, median (IQR), °C	37.2 (36.9-37.7)	37.2 (36.8-37.6)
Therapy in the ED		
Oxygen	2 (1.1)	4 (2.2)
Intravenous fluids	3 (1.6)	0
Inhaled epinephrine	15 (8.2)	14 (7.7)
Inhaled albuterol	17 (9.2)	18 (9.8)
Dexamethasone	12 (6.5)	15 (8.2)
Length of ED stay, median (IQR), h	4.1 (2.9-6.0)	4.0 (2.7-5.1)

Abbreviation: ED, emergency department.

^a^ Refers to history of atopic dermatitis in participants.

^b^ Refers to history of allergic rhinitis, asthma, or allergic dermatitis in parents or siblings.

Overall, 182 participants (49.6%) received suctioning exclusively via the assigned device (per-protocol treatment): 106 in the enhanced suction group and 76 in the minimal suction group. Of the 185 children not treated per protocol, 108 (58.4%) were in the minimal suction group (97 parents used additional nose-to-mouth suctioning devices, 7 used battery-operated tools, and 4 did not suction), and 77 (41.6%) were in the enhanced suction group (68 parents also used nose-to-mouth devices, 8 used bulb, and 1 used an additional battery-operated tool). A total of 165 of 185 families (89.2%) not treating per protocol used mouth-to-nose devices as additional suctioning tools: 136 of 165 families (73.5%) also used mouth-to-nose devices before arrival. Missing data were minimal (19 patients). Nonimputed data were used in the presented results.

### Primary Outcome

In the trial, 116 of 367 participants (32.7%) used additional resources, including 68 of 184 (37.0%) in the minimal suction group vs 48 of 183 (26.2%) in the enhanced suction group (absolute risk difference, 0.11; 95% CI, 0.01 to 0.20; *P* = .03). Unscheduled visits occurred for 47 of 184 infants (25.5%) in the minimal suction group and for 40 of 183 infants (21.9%) in the enhanced suction group (absolute risk difference, 0.04; 95% CI, −0.05 to 0.12; *P* = .46). The per-protocol analysis confirmed comparable revisits between the groups (Table 2).

**Table 2.** Primary Outcome of Additional Resource Use and Its Components

Outcome	Participants, No. (%) (N = 367)		Absolute risk difference (95% CI)	*P* value	Adjusted risk difference (95% CI)^a^	*P* value
	Minimal suction group (n = 184)	Enhanced suction group (n = 183)				
Additional resource use^b^	68 (37.0)	48 (26.2)	0.11 (0.01 to 0.20)	.03	0.12 (0.02 to 0.21)	.01
Unscheduled visits by 72 h	47 (25.5)	40 (21.9)	0.04 (−0.05 to 0.12)	.46	0.04 (−0.04 to 0.13)	.32
Unassigned device use for lack of feeding or breathing improvement	33 (17.9)	11 (6.0)	0.12 (0.05 to 0.18)	<.001	0.12 (0.05 to 0.19)	<.001
Per-protocol analysis of unscheduled visits, No./total No. (%)^c^	15/76 (19.7)	22/106 (20.8)	−0.01 (−0.13 to 0.11)	>.99	−0.01 (−0.11 to 0.10)	.87

^a^ Post hoc adjustment was made for stratification at randomization for age group, with site as a random effect.

^b^ Defined as either an unscheduled visit to any health care facility for bronchiolitis within 72 hours of ED discharge or the use of additional suctioning devices for perceived feeding and/or breathing problems.

^c^ This was a sensitivity analysis with patients using the assigned device only.

A total of 33 of 184 infants (17.9%) in the minimal suction group were suctioned with additional devices because of a perceived lack of feeding or breathing improvement vs 11 of 183 infants (6.0%) in the enhanced suction group (absolute risk difference, 0.12; 95% CI, 0.05-0.19; *P* < .001). Adjusted analyses showed comparable results (Table 2).

### Secondary Outcomes

We found no significant between-group differences in the proportions of all bronchiolitis-related revisits (absolute risk difference, 0.07; 95% CI, −0.02 to 0.16; *P* = .15), ED revisits (absolute risk difference, 0.04; 95% CI, −0.03 to 0.12; *P* = .30), parental satisfaction with care at home (absolute risk difference, −0.02; 95% CI, −0.10 to 0.06; *P* = .70), and in normal infant feeding (difference in differences, 0.03; 95% CI, −0.10 to 0.17; *P* = .62) and sleeping (difference in differences, 0.05; 95% CI, −0.08 to 0.18; *P* = .47) at 72 hours (Table 3). The proportion of parents reporting their own sleep as normal was higher in the enhanced group but the difference was not statistically significant (difference in differences, 0.10; 95% CI, −0.02 to 0.23; *P* = .09) (Table 3). The differences of the changes in infant feeding and sleeping adequacy and parental sleep adequacy over time were not significant (Table 4). The per-protocol analyses of the secondary outcomes yielded results comparable to those of the intention-to-treat results (eTable 1 and eTable 2 in Supplement 2).

**Table 3.** Secondary Outcomes of the Trial

Outcome	Participants, No. (%) (N = 367)		Absolute risk difference (95% CI)	*P* value	Adjusted risk difference (95% CI)^a^	*P* value
	Minimal suction group (n = 184)	Enhanced suction group (n = 183)				
All medical revisits^b^	53 (28.8)	40 (21.9)	0.07 (−0.02 to 0.16)	.15	0.08 (−0.01 to 0.16)	.08
ED revisits	31 (16.9)	23 (12.6)	0.04 (−0.03 to 0.12)	.30	0.05 (−0.02 to 0.11)	.16^c^
Normal feeding at 72 h^d^	112 (60.9)	122 (66.7)	−0.06 (−0.16 to 0.04)	.28	−0.06 (−0.15 to 0.04)	.25
Normal sleep at 72 h	86 (46.7)	86 (47.0)	−0.003 (−0.10 to 0.10)	>.99	−0.003(−0.11 to 0.10)	.95
Normal parental sleep	51 (27.7)	71 (38.8)	−0.11 (−0.21 to −0.02)	.03	−0.11 (−0.21 to −0.02)	.02
Satisfied with care after ED discharge^e^	145 (78.8)	148 (80.9)	−0.02 (−0.10 to 0.06)	.70	−0.02 (−0.11 to 0.06)	.58

Abbreviation: ED, emergency department.

^a^ Post hoc adjustment was made for stratification at randomization for age group, with site as a random effect.

^b^ Defined as any unscheduled visit for bronchiolitis to any medical facility or practitioner within 72 hours of ED discharge.

^c^ Based on logistic regression analysis.

^d^ Defined as greater than 80% normal fluid intake.

^e^ Defined as satisfied or very satisfied with bronchiolitis care after discharge.

**Table 4.** Changes in Feeding and Sleeping During the Trial

Outcome	Minimal suction group (n = 184)			Enhanced suction group (n = 183)			Unadjusted		Adjusted^a^	
	Participants, No. (%)		Unadjusted difference (95% CI)	Participants, No. (%)		Unadjusted difference (95% CI)	Difference-in-differences (95% CI)	*P* value	Difference-in-differences (95% CI)	*P* value
	Baseline	72 h		Baseline	72 h					
Normal feeding^b^	52 (28.3)	112 (60.9)	0.33 (0.23 to 0.42)	56 (30.6)	122 (66.7)	0.36 (0.27 to 0.46)	0.03 (−0.10 to 0.17)	.62	0.03 (−0.10 to 0.17)	.64
Normal sleep	31 (16.9)	86 (46.7)	0.30 (0.21 to 0.39)	40 (21.9)	86 (47.0)	0.25 (0.16 to 0.35)	0.05 (−0.08 to 0.18)	.47	0.04 (−0.09 to 0.17)	.50
Normal parental sleep	27 (14.7)	51 (27.7)	0.13 (0.05 to 0.21)	28 (15.3)	71 (38.8)	0.24 (0.15 to 0.32)	0.10 (−0.02 to 0.23)	.09	0.11 (−0.01 to 0.23)	.07

^a^ Post hoc adjustment was made for stratification at randomization for age group, with site as a random effect.

^b^ Defined as greater than 80% normal fluid intake.

### Other Outcomes

Most caregivers using the enhanced suction were satisfied with their assigned device (145 of 183 caregivers [79.2%]) compared with their minimal suction counterparts (62 of 184 caregivers [33.7%]; risk difference, 0.45; 95% CI, 0.36-0.54; *P* < .001). The most common reasons for dissatisfaction with minimal suctioning were perceived poor suctioning (80 caregivers), irritability (34 caregivers), and cumbersome device use (33 caregivers) vs poor suctioning (19 caregivers), irritability (5 caregivers), and cumbersome use (4 caregivers) in the enhanced suction group. The per-protocol analysis confirmed that 38 of 76 parents (50%) using minimal suctioning only were satisfied with their device vs 101 of 106 (95.3%) of their compliant enhanced suction counterparts (difference, –0.45; 95% CI, −0.33 to −0.57; *P* < .001).

### Adverse Events

A total of 111 participants (30.2%) experienced disease-related expected adverse events, including 67 (36.4%) in the minimal suction group and 44 (24.0%) in the enhanced suction group. The most common event was hospitalization after ED discharge (18 of 367 patients [4.9%]: 11 of 184 [6.0%] in the minimal suction group and 7 of 183 [3.8%] in the enhanced suction group).

Unexpected adverse events occurred in 2 infants in each group who had a nosebleed leading to a medical visit. Neither infant required any intervention, and both events were judged as possibly device-related but mild. Five infants were admitted to intensive care unit (2 in the minimal suction group and 3 in the enhanced suction group); all recovered and none of the events was judged as being device related.

## Discussion

In this randomized clinical trial of infants with bronchiolitis discharged home from the ED, enhanced suctioning did not alter the disease course compared with minimal suctioning, as demonstrated by comparable rates of bronchiolitis-related revisits within 72 hours after discharge. The proportions of infants with normal feeding and sleeping at 72 hours also did not differ, supporting the lack of effect of enhanced suctioning on bronchiolitis severity. The observed difference in the additional resource use was almost entirely associated with a substantial between-group difference in the use of nonassigned devices due to parent-perceived lack of feeding or breathing improvement.

A retrospective study^25^ of infants hospitalized for bronchiolitis reported that suctioning lapses of more than 4 hours were associated with longer length of hospital stay. However, that study focused on inpatients and investigated both nasal and deep suctioning. A recent randomized ED study^26^ of infants discharged home with bronchiolitis found no difference in revisits within 14 days in those suctioned by a bulb vs a nasal-oral aspirator. That study was limited by a single-center design, lack of a reference standard, and substantial follow-up loss. In contrast, our multicenter randomized clinical trial used suctioning interventions pretested for their plausible mechanistic benefit, and the enhanced intervention was standardized to mirror in-hospital suctioning.

Our study demonstrates that enhanced suctioning after discharge does not yield fewer unscheduled revisits than minimal suctioning. The most important reason is that enhanced suctioning does not alter the disease course. The study infants were suitable for discharge home and had mild disease, which likely also contributed to the observed lack of incremental benefit of enhanced suction on feeding and sleeping adequacy. Second, we excluded infants with comorbidities, who represent a high-risk category for more severe outcomes.^39^ Although bronchiolitis is an anxiety-provoking condition with a high revisit burden,^5,32,40^ these revisits rarely result in management changes.^5^

Minimal suctioning resulted in a significantly higher rate of additional device use than enhanced suctioning. The bulb allows minimal time for negative pressure generation, which likely yielded low suctioned volumes, as confirmed in the questionnaire where poor suctioning represented the majority of reasons for dissatisfaction with minimal suctioning. For this and other reasons, the parents in this group were less satisfied with the device than those assigned to the enhanced battery-operated device with high negative pressures, and they, thus, resorted to additional devices. In addition, more than one-half of the families were using mouth-to-nose devices before arrival, so those assigned to minimal suctioning were understandably motivated to upgrade the suction mode with the devices previously used. Indeed, almost all noncompliant parents resorted to the mouth-to-nose devices and three-quarters of them had used these tools previously. This study highlights parental tendency to do something, and this was particularly true of the parents using minimal suctioning. There is a physician bias for perceived benefit of medications they prescribe,^41^ and the same may be true of parents using their favorite mouth-to-nose suctioning devices, which were previously shown to yield revisit rates comparable to those for the minimal suctioning.^26^

The lack of incremental benefit of enhanced suctioning in changing bronchiolitis course should provide physicians with reassurance in addressing parental fear about their infant’s potential for having worse outcomes while doing less in bronchiolitis. Parents should also be counseled that enhanced suctioning comes at a cost: battery-operated suctioning devices are substantially more costly than the bulb (approximately $45 vs $5, respectively).

### Limitations

This study has limitations. Parents could not be blinded to the intervention, which affected their compliance with the study protocol and may have affected some of the outcomes. However, the per-protocol analyses confirmed a lack of between-group differences in the bronchiolitis progression between groups. Because the 95% CI around the difference in the primary outcome in the per-protocol analysis includes 15%, the study was underpowered to detect small differences. In the current nasal suctioning–oriented practice milieu, it was not feasible to include a group with no suctioning. Therefore, although our prestudy work showed that the bulb provides minimal suctioning, the results may not be fully applicable to no suctioning. In addition, the results of this study are not generalizable to suctioning practices in the ED or in the inpatient setting.

## Conclusions

Enhanced suctioning after ED discharge with bronchiolitis did not alter the disease course compared with minimal suctioning. Minimal suctioning yielded significantly higher use of nonassigned suctioning devices with a lower parental satisfaction with the assigned device than the enhanced approach.

## Notes

Supplement 1.
Trial Protocol and Statistical Analysis Plan

Supplement 2. **eAppendix 1.** Follow-Up Questionnaire **eAppendix 2.** Feeding and Sleeping Adequacy Questions **eAppendix 3.** Satisfaction With Care After Discharge **eTable 1.** Secondary Outcomes of the Trial: Per-Protocol Analyses **eTable 2.** Changes in Feeding and Sleeping During the Trial: Per-Protocol Analyses

Supplement 3.
Pediatric Emergency Research Canada (PERC) Network Members

Supplement 4.
Data Sharing Statement
