# Supplementary material for: Nasal Suctioning Therapy Among Infants With Bronchiolitis Discharged Home From the Emergency Department: A Randomized Clinical Trial
Source: JAMA Netw Open. 2023 Oct 19;6(10):e2337810. doi: 10.1001/jamanetworkopen.2023.37810 (PMC10587796; doi:10.1001/jamanetworkopen.2023.37810)
Supplement: Supplement 1. — Trial Protocol and Statistical Analysis Plan [file jamanetwopen-e2337810-s001.pdf]

Original Protocol

Suctioning of NOse Therapy (SNOT) versus Usual Home Care in Bronchiolitis - a Randomized Clinical Trial

Principal Investigator:

Dr. Suzanne Schuh The Hospital for Sick Children, Toronto, Ontario

Co-Investigators:

Dr. Yaron Finkelstein The Hospital for Sick Children, Toronto, Ontario

Dr. Patricia Parkin The Hospital for Sick Children, Toronto, Ontario

Dr. Allan Coates The Hospital for Sick Children, Toronto, Ontario

Dr. Andrew Willan The Hospital for Sick Children, Toronto, Ontario

Dr. Amy Plint The Children's Hospital of Eastern Ontario, Ottawa, Ontario

Dr. Roger Zemek The Children's Hospital of Eastern Ontario, Ottawa, Ontario

Dr. Naveen Poonai London Health Sciences Centre, London, Ontario

Dr. Mohamed Eltorki McMaster Children's Hospital (Hamilton Health Sciences), Hamilton, Ontario

Collaborator:

Diane Soares RRT The Hospital for Sick Children, Toronto, Ontario

BACKGROUND

Burden of bronchiolitis: Bronchiolitis is a viral syndrome of infants under 12 months of age, characterized by the first episode of rhinorrhea, cough and respiratory distress[1]. Bronchiolitis represents a disease with low mortality rates but very high morbidity; it is the leading cause of infant hospitalizations in the Western world, with increasing costs over the past several decades.[1-3] In the United States, bronchiolitis-related annual hospitalization expenses exceed $500 million,[4,5] with similar high economic burden in other Western countries.[6] More than one third of all infants develop bronchiolitis within the first year of life, of whom 3% are admitted to hospital.[7] Of infants diagnosed with bronchiolitis in the Emergency Departments (ED), 30-40% requires hospitalization.[8] The reported annual healthcare utilization rate for bronchiolitis per 1000 infants under 6 months of age is 17 hospitalizations, 55 ED visits, and 132 unplanned office visits.[3] Return visits are also commonplace: a large prospective multicenter study demonstrated that 20% of infants discharged home with bronchiolitis from the Emergency Departments (ED) in the United States have unscheduled visits for persistent or worsening symptoms within the subsequent 2 weeks.[9] Therefore, bronchiolitis is an extremely common and expensive problem, with considerable impact on families!

Challenges with management of bronchiolitis: Despite extensive research, current principles for bronchiolitis management remain similar to those recommended half a century ago.[10] Since effective therapies for bronchiolitis remain elusive, the American Academy of Pediatrics (AAP), Canadian Paediatric Society and other professional societies advocate for the use of supportive measures such as adequate oxygenation, hydration and airway stabilization and discourage routine use of pharmacotherapy.[1,11-16] The paucity of effective evidence-based therapy has led to substantial practice variation, including use of unwarranted treatments.[17-26] Indeed, management of bronchiolitis is more strongly associated with local hospital practices or individual physician preferences than with disease severity[27].

Since guidelines recommend offering only supportive care to infants with bronchiolitis, it is important to determine which aspects of supportive care are effective. While little doubt exists about the need for intravenous rehydration in lethargic, dehydrated infants and for supplemental oxygen in infants with respiratory distress and significant hypoxemia, no previous study has examined the benefits of nasal suctioning in bronchiolitis.

Importance of Nasal Patency in Bronchiolitis: An important challenge associated with caring for patients with bronchiolitis concerns the fact that it involves young infants less than one year of age. Young infants are obligate nose breathers[28] and their inability to remove nasal secretions by nose blowing contributes to bronchiolitis morbidity such as augmented respiratory distress, altered sleep cycle and feeding difficulties.[28,29] Maintaining hydration is critically important in bronchiolitis care: poor feeding associated with respiratory distress represents a major reason for admitting babies with bronchiolitis to hospital.[28]

Although bronchiolitis involves primarily the lower respiratory tract, dysfunction of the upper airway also plays a key role in the pathogenesis, with nasal edema and inflammatory exudate as its integral components. Furthermore, as much as 50% of the total airway resistance originates in the nose which plays a key role in humidification of inhaled air, ciliary activity as well as in providing innate immunity and modulation of inflammation.[28,30,31]

What is known about nasal suctioning in bronchiolitis? Although there is no proven therapy to ameliorate the lower airway pathology in bronchiolitis, many practitioners employ supportive measures related to relieving nasal obstruction. Specifically, a study by Mallory which examined common treatment strategies in bronchiolitis employed by 519 pediatricians in the United States reported that 82% of the responders utilized nasal suctioning as the most common therapeutic strategy and this proportion exceeded the frequency of all other treatment approaches.[32] The authors emphasize that *this commonly used intervention is not evidence-based and needs to be further explored.*[32]

Based on intuitive practice rather than on proven evidence, several comprehensive bronchiolitis guidelines from North America, Europe and Australia as well as some reviews

recommend consideration of nasal suctioning as a therapeutic option. These include the NICE guideline from the U.K., the Canadian Pediatric Society Guideline, the Scottish Intercollegiate Guidelines Network (SIGN), the inter-society consensus document from Italy, Clinical Practice Guidelines in the Spanish National Healthcare System, and the HUGO guideline from France.[12-14,33-37] The SIGN and American Academy of Pediatrics (AAP) 2014 guidelines highlight the expert consensus as the basis for this recommendation, and point out lack of relevant evidence supporting its benefit.[1,13] Literature also suggests plausible but unproven hypotheses that the transcutaneous oxygen saturation should be measured after nasal aspiration.[28,38] A recent parental survey found that 90% of parents perceive nasal suctioning as effective in improving infants' symptoms.[39] Given how frequently suctioning is employed, *the benefits of this commonly employed intervention need to be established before this treatment modality can be recommended.* Furthermore, although the widespread superficial method to be used in this study of nasal suctioning is generally regarded to be safe, the side-effects of this intervention have not been systematically documented. Some healthcare providers find that infants sometimes perceive nasal suctioning as unpleasant (see also Safety section) and we shall thus track adverse effects of suctioning during the study. Therefore, even if the study yields negative results, this would provide important evidence and inform clinical care.

Suctioning device:

In a study of nasal suctioning, it is imperative to implement an effective suctioning tool. We have investigated several such devices available on the Canadian market. The main disadvantage of the oral suctioning units (where the caretaker suctions the nose) such as Hydrasense (Shering Plough Canada), Naspira (NeilMed Pharmaceuticals) and Nosefrida is their operator effort- dependence and consequent variability in the generated negative pressures and aspirated volumes. Furthermore, we found that the aspirated fluid consistently crossed the filter barrier, posing a potential infection safety issue to the operator. The ubiquitous commonly used bulb aspirators are limited by a very short time interval of ≤ 1 second available for bulb release during aspiration which results in non-sustained aspiration pressures. The Babyvac device (Illes Csok Es TSA, Budapest, Hungary) has to be attached to a vacuum cleaner

which may prevent many parents from using it. This device is also noisy and generates variable pressures, depending on the vacuum model.

In contrast, the battery-operated device by Zo-Li (Zo-Li, Inc, CA, U.S.A.) generates consistent and reproducible high negative pressure of 126 mm Hg which is sustained over the 10 second suctioning interval. This pressure is comparable to the 120 mm Hg pressure which represents the accepted Canada-wide hospital standard for the wall suction available in the EDs and other hospital settings. The interface is a nasal cushion that makes a tight but comfortable seal at the nares. An important advantage of the Zo-Li device over other available suctioning tools is that is operator effort- independent and has consistent performance. Importantly, it is available for purchase in Canada with detailed instructions for safe use.

Research Questions:

<u>Primary:</u> In otherwise healthy infants 4 weeks to 12 months of age discharged home from the ED with acute bronchiolitis,[1] is there a difference in the probability of "treatment failure" by day 7 post index ED visit in those receiving enhanced nasal suctioning via Zo-Li device prior to feeds compared to infants who receive usual care? Treatment failure is defined as any bronchiolitis-related healthcare visit, except visits that are ED-recommended at time of ED discharge (see Outcome Measures section) within 7 days post discharge at the index ED visit.

<u>Hypothesis:</u> We hypothesize that the infants who undergo enhanced scheduled nasal suctioning will experience a lower treatment failure probability by day 7 post discharge from index ED visit compared to those managed by usual care.

<u>Secondary:</u>

1- In these infants, is there a difference in the mean number of unscheduled medical visits for bronchiolitis within 7 days of ED discharge?
2- In these infants, is there a difference in the mean number of ED visits for bronchiolitis within 7 days of ED discharge?

3- In these infants, is there a difference in the probability of a parent reporting normal/near normal feeds on approximately day 7 post ED discharge?
4- In these infants, is there a difference in the probability of a parent reporting normal/near normal sleeping on approximately day 7 post ED discharge?
5- For the parent, is there a difference in the probability of a parent reporting their own normal/near normal sleeping on approximately day 7 post ED discharge?
6- In these infants, is there a difference in the probability of parents reporting on day 7 post ED discharge as being "very satisfied" or "satisfied" with their ability to care for their child during the study period?

Study Design:

This is a four-center, randomized, outcome assessor- blinded clinical trial of infants discharged home from the ED with acute bronchiolitis. Two groups will be compared: infants receiving usual bronchiolitis care (see below) during the 7 days post ED discharge versus those given usual care plus enhanced nasal suctioning via a standardized nasal aspiration Zo-Li device just prior to each feeding for 7 days post index ED visit discharge. Evidence suggests that suctioning at frequent intervals is associated with movement of secretions from the lower airway with improved minute volume.[40] The 7- day study period has been chosen since the vast majority of infants discharged from the ED with bronchiolitis experience the targeted outcomes within this time frame.[9]

Prior to the ED discharge, all study participants will receive usual bronchiolitis care ordered by the ED physician as per standard of care which consists of supplemental oxygen for saturations <90% and supplemental IV hydration in those with inadequate oral intake.[41] Eligible consenting patients deemed suitable for discharge home (representing approximately 60-70% of the bronchiolitis population presenting to the ED) will be randomly allocated to one of two study groups:

<u>Control Group:</u> this group will receive standardized routine discharge instructions describing information about bronchiolitis, expected course of illness, recommended management

strategies such as fever control, augmented air humidification, need for frequent feeding and warning signs prompting return for care. Some ED treating physicians also recommend removal of nasal secretions via dropper attached to a bulb (usual strategy which produces negligible negative pressures) or via other commercially available aspirators (used less commonly) - this decision will be left up to the treating ED physician and the use of such devices will be tracked. This study design is in keeping with the pragmatic nature of the study used to show the real-world differences of the intervention.[42] Our clinical experience suggests that the Zo-Li device is currently not commonly used. Furthermore, we shall not reveal the identity of the experimental device to the ED physicians in order to minimize contamination of the control group.

Experimental Group: in addition to the aforementioned usual bronchiolitis care at home, this group will undergo nasal suctioning prior to each feeding as needed for 7 days post discharge home (unless symptoms completely abate earlier), using exclusively the Zo-Li study device (see above under study device), with saline nose drops if needed. Families in this group will be given the Zo-Li device at no cost and instructed in the appropriate technique and importance of using this tool. This explanation may enhance parental motivation to use the device.

Study Population:

Inclusion criteria:

1- Diagnosis of bronchiolitis in the ED as per the AAP diagnosis definition. [1]Bronchiolitis is defined as the first episode of viral upper respiratory infection with respiratory distress and/or tachypnea for age.
2- Age 4 weeks up to and including 12 months of age. Participating infants will have to be at least 4 weeks post their expected due date of birth since infants with bronchiolitis below this age cut-off are at a much higher risk of apnea and dehydration than their older counterparts.[43]

3- Below usual feeding intake in the past 24 hours. This will be screened by the study nurse who will ask the caregiver(s) the following questions: Compared to usual, how has your infant fed in the last 24 hours?
   a. Definitely less than normal but more than half usual intake or some difficulty breastfeeding[16]
   b. Very much less than normal (less than half usual intake or major difficulty breastfeeding[16]
4- Must have at least <u>one</u> of the following: home/cellular telephone or e- mail
5- Informed consent

Exclusion criteria:

1. Previous diagnosis of bronchiolitis made more than 3 weeks prior to this ED visit.[44]
2. Hospitalization at the index ED visit. Although we have considered starting the experimental intervention at presentation to the ED, this was deemed counterproductive since many patients with bronchiolitis are currently routinely suctioned in the ED which would likely contaminate the control arm and impact study results.
3. Normal/near normal feeding in the past 24 hours. The feeding adequacy will be screened by the study nurse who will ask the caregiver(s) the following questions:
   Compared to usual, how has your infant fed in the last 24 hours?
   Normal/ almost normal [more than about 80% usual intake]
4. Zo-Li battery operated nasal aspirator used at home.
5. Co-morbidities which may impact outcomes such as known diagnosis of congenital heart disease, chronic respiratory disease including known lung disease due to prematurity, aspiration due to severe gastro-esophageal reflux, neuro-muscular/neurologic disease, immunodeficiency, coagulopathies, nasal/upper airway abnormalities, oral, gastrointestinal anomalies [except for corrected pyloric stenosis], tracheo-esophageal fistulas, gastric/gastro-jejunal tube feeding supplementation.
6. Parental poor command of the English/French language.

Sample selection:

Infants presenting to the collaborating four EDs at the Hospital for Sick Children, the Children's Hospital of Eastern Ontario, London Health Sciences Centre and McMaster Children's Hospital who meet eligibility criteria will be approached for enrollment when the research nurse/research assistants are on duty (days and evenings). The research nurse/research assistants will keep a log of all infants presenting to the ED with bronchiolitis during the study period whether randomized or not in order to confirm lack of enrollment bias. Specifically, information collected in non-enrolled patients will include age, gender and disposition from the ED. All of the aforementioned hospitals are paediatric tertiary care centers which see the entire clinical and demographic spectrum of the bronchiolitis population. Our profile of children with bronchiolitis is therefore comparable to that of other institutions, the generalizability of the study should not be affected and referral bias should be minimal. A standardized data collection form will be used to assess the baseline and demographic features that may affect outcome and potentially confound the comparisons. Since the patients will be screened consecutively and study coverage will occur during days, evenings and weekends, selection bias should not play a significant role.

Allocation to Study Groups:

Sequence generation: Data management department at the Research Institute at the Hospital for Sick Children will provide data management services for this study. In this study, we shall use a third-party treatment allocator, the randomization service www.randomize.net for treatment assignment. We have successfully used this service in previous trials. This service will produce and securely guard a randomization list produced with the help of random-number generating software. *Allocation concealment*: This randomization service will allocate eligible consenting patients to the two study groups, with the assignment stratified by site and age (< 6 months vs ≥ 6 months), using a permuted block randomization of 4 and 6 with a 1:1 allocation ratio. The allocation sequence will be securely stored at the randomization service site until study enrollment has been concluded and the analyses have been finalized. *Implementation:*

Upon receiving the e- mail indicating the study number and group assignment of a given participant, the study nurse will obtain the next consecutively numbered study kit for that patient and enter the number into a confidential log book. The kits will contain standardized bronchiolitis discharge instructions which will be supplemented with the Zo-Li suctioning device and written instructions on its use for infants assigned to the experimental intervention.
The study kits, the Zo-Li interventional device and the logbook will be kept in a locked research cabinet in the ED.

Minimizing bias:

Bias will be minimized by strictly adhering to the 2010 CONSORT Statement recommendations including the use of "third-party" treatment assignment.[45] In this study, outcome adjudicators, i.e. research assistants performing follow up and the data analyst will be blinded to the study hypothesis and to the intervention and the ascertainment bias will thus be minimized. The question on home suctioning during telephone/e-mail follow up will inquire about the use of bulb, oral, vacuum-attached, and battery-operated suction but the assistant will not know that the Zo-Li device is under scrutiny. Although the assistant may become unblinded due to frequent use of Zo-Li, this should not introduce a significant bias as this will be the last question during contact with the parents. The ED treating physicians will also be blinded to treatment assignment and to the device under study in order to minimize contamination of the control group. For ethical and practical reasons, it is not feasible to blind parents to study intervention. However, the primary outcome (bronchiolitis-related medical visits and hospitalizations) is a health care utilization variable not likely to be impacted by lack of parental blinding. An intention-to-treat analysis will be performed to minimize bias associated with poor compliance and non-random loss of participants.[46] Co-interventions (e.g. use of saline nasal drops) and other sources of confounding will be recorded. Reporting bias will be avoided by registering the study at clinicaltrials.gov. To blind the study analyst to treatment allocation, the assigned allocation in the electronic study database will be designated only by an unidentified "group A" or "group B" designation.

Referral bias is unlikely since all participating EDs see patients with the entire spectrum of bronchiolitis severity and the results should be also generalizable to the general EDs. Furthermore, the Children's Hospital of Eastern Ontario, the London Health Sciences Centre, London Ontario, and the McMaster Children's Hospital are the only pediatric inpatient facilities in the region and The Hospital for Sick Children serves as a community hospital for downtown Toronto and also is used by many families who reside within its large catchment area. The patients will be screened consecutively and study coverage will occur days, evenings and weekends. The study nurse/research assistants will keep continuous study logs with the characteristics including age, gender and vital signs in triage of the participating and non-participating patients which will also track missed patients, those excluded for criteria and patients who refused participation to document any selection bias.

Pre-study screening and baseline evaluation

All previously healthy infants 4 weeks to 12 months of age with the first episode of respiratory distress and upper respiratory infection will have vital signs and oxygen saturation assessed by the triage staff, as per usual clinical routine and will undergo treatment as per local standard of bronchiolitis care.

The research nurse/research assistants on duty will pre-screen potentially eligible patients for age and for respiratory complaints by reviewing the Electronic Data Information System triage note and by communication with the treating physician as to the confirmed diagnosis of bronchiolitis. Once permission to be approached for research has been obtained from the caretakers using site specific and privacy office approved approach methods, the research nurse/research assistants on duty will assess eligibility and obtain written informed consent after planned discharge home has been communicated. Thereafter, the research nurse/research assistants will document relevant demographic and clinical information.

Study procedure:

1- The eligible consented participants will be randomly allocated by www.randomize.net to the two study groups. All participants will be given standardized bronchiolitis discharge

instructions according to each individual site regarding anticipated duration of symptoms of up to 2-3 weeks,[47] need for small, frequent feeds and fever control, and encouragement to return to ED if there is marked decrease in current feeding pattern, inappropriate lethargy, poor urine output/no wet diapers in 12 hours, more labored/rapid breathing. Those assigned to the experimental group will also be provided with the Zo-Li suctioning device and instructed on its use and care.

2- Prior to discharge home, the study nurse will measure the vital signs, neck/chest retractions and transcutaneous oxygen saturation as per usual routine, to document comparable disease severity in the groups. To ensure comparable nasal congestion status in both study groups, all participants will be nasally suctioned with the ED wall suction prior to discharge. In addition to the aforementioned eligibility screening question about preceding feeding adequacy, the research nurse will also inquire about their child's and their own sleeping adequacy over the past 24 hours by asking the following Likert scale questions: 1) "In the past 24 hours, how has your child slept compared to usual?" a) very much less than normal, b) less than normal), c) normal/almost normal d)somewhat more than normal e) very much more than normal 2) "In the past 24 hours, how have <u>you (the caregiver)</u> slept compared to usual?" a) very much less than normal b)somewhat less than normal c) normal/almost normal d)somewhat more than normal e) very much more than normal

3- There will be a telephone/e- mail follow- up of all randomized patients on approximately day 7 by a research assistant trained in interviewing techniques and blinded to the study hypothesis and intervention assignment to determine subsequent hospitalization, family and physician-initiated medical visits, the level of feeding and sleeping adequacy and the level of perceived parental satisfaction to care for their child's illness.

Also, the use of any suctioning devices will be recorded, as will be information about the potential adverse effects due to suctioning such as nose bleeding, prolonged screaming >5

minutes after suctioning, vomiting, and increased respiratory distress. We shall also ask about parental satisfaction with the suctioning device (any) used by asking the following Likert scale questions: 1) “During the past week, I have used device “x” to suction my child’s nose. The following best describes my satisfaction with this device: a) very satisfied, b) satisfied, c) neither satisfied nor dissatisfied, d) dissatisfied, e) very dissatisfied, f) not applicable- no suction tool used”. The assistant will also ask the dissatisfied parents a qualitative question about what their reasons for dissatisfaction were such as associated noise from the device, inconvenience, excessive irritability of the infant, side effects listed above, 2) “Please tell us about your agreement with using the same suctioning tool again”: a) very much agree, b) agree, c) neither agree not disagree, d) disagree, e) very much disagree, f) not applicable. These responses will be used in a descriptive rather than analytical manner.

Outcome Measures

The primary outcome measure will be a composite outcome of “treatment failure” due to bronchiolitis symptoms, defined as any bronchiolitis-related healthcare visit, except visits that are ED-recommended at time of ED discharge within 7 days post discharge at the index ED visit. This visit will include either 1) hospitalization for bronchiolitis to any inpatient facility or 2) family-initiated (unscheduled) bronchiolitis-related medical visits with any health care provider or 3) physician-initiated visits for bronchiolitis within 7 days of discharge (such as the re-visits suggested by the primary care provider), excluding the visit recommended only by the ED treating physician. All components of this outcome reflect concern about respiratory distress or inadequate feeding. In our past bronchiolitis study, we have determined that a considerable portion of infants with bronchiolitis consume these resources.[48] Bronchiolitis symptoms are most severe and impactful during the first week of illness,[49] about a third of infants with bronchiolitis discharged from the EDs have subsequent bronchiolitis-related visits[50] and the overwhelming majority of these occur within the study time frame.[9]

This outcome is of importance to the families due to the stress and inconvenience of subsequent return visits or hospitalization. It quantifies the morbidity burden of ongoing

bronchiolitis symptoms and is also related to financial setback and health care dollars investment. Health care utilization represents a powerful marker of bronchiolitis morbidity that has the capacity to alter clinical practice and influence decision makers.

Secondary outcomes:

1- Family-initiated bronchiolitis-related medical visits within 7 days of the index ED visit. We anticipate that patients in the interventional group may have fewer/milder symptoms and less parental anxiety, which may lead to fewer unscheduled visits. In infants discharged from the ED for bronchiolitis, approximately 37% have future family-initiated visits for bronchiolitis during the course of illness which constitute more than a half of all return visits.[51]

2- Emergency Department visits for ongoing/worsening bronchiolitis symptoms within 7 days of the index ED visit. Patients returning to/referred to the ED represent the most severe end of the disease spectrum and a difference in this outcome will therefore reflect a clinically important difference in symptoms.

3- Normal/near normal feeding on day 7. This will be ascertained by the research assistant/research nurse by asking the following question at the e- mail/telephone interview on approximately day 7 at the time mutually agreed upon prior to the ED discharge: “Compared to usual, how much food has your infant taken in the last 24 hours? a) normal/near- normal amount [more than about 80% normal]), b) definitely less than normal but more than about 50% normal amount/some difficulty breastfeeding c) very much less than normally (less than 50% normal/major difficulty breastfeeding[16]

4- Normal/near normal sleeping adequacy on day 7. This will be ascertained by the research assistant/research nurse by asking the following question at the telephone/e-mail interview on approximately day 7: “Based on my child’s sleeping pattern, his/her sleep in the past 24 hours was the following compared to usual: a) very much less than normal b) somewhat

less than normal c) normally/almost normally), d) somewhat more than normal), e) very much more than normal

5- Parental normal/near normal sleeping adequacy on day 7 post discharge. This will be based on the question: "In the past 24 hours, how have you (the caregiver) slept compared to usual?" a) very much less than normal b) somewhat less than normal c) normally/almost normally d) somewhat more than normal e) very much more than normal
6- Parental level of satisfaction with their ability to care for their child's illness within the 7 days of discharge from the ED. This will be ascertained by the research assistant/research nurse asking the following question on day 7: "Since discharge from the ED, I would rate my level of satisfaction with my ability to care for my child's illness as follows: a) very satisfied, b) satisfied, c) neither satisfied not dissatisfied, d) dissatisfied, e) very dissatisfied." This outcome will reflect the difference in the level of perceived parental ability to provide the needed comfort to their offspring in the two study groups.

Other outcomes: Clinically important side-effects such as prolonged crying with the use of any suctioning device for more than about 5 minutes, epistaxis, vomiting and increased respiratory distress will be tracked. These outcomes are uncommon and the study cannot reasonably be powered for their meaningful analysis. However, these data are important to establish an estimate of a safety profile of the study intervention and of the usual practice.

Outcome Measures Measurement

Baseline (pre-discharge) measurements: The pre-discharge vital signs and saturation will be measured in the ED by the study nurse/research assistants as per usual practice. They will also document the duration of respiratory distress prior to ED arrival, pre-arrival feeding and sleeping status of the patients and review the *ED electronic data records* to confirm subsequent visits for bronchiolitis by day 7 to our ED. Trained research assistants will obtain information about subsequent hospitalizations, family-initiated medical visits for bronchiolitis and feeding/sleeping adequacy of the participants during the standardized *telephone/e- mail follow-ups* on day 7. If the families cannot be reached on this day, daily phone calls/e- mails will be made for the next 7 days

Sample Size

The sample size calculation is based on the assessment of the between-group difference in probability of treatment failure. The estimated total re-visit probability in bronchiolitis based on a recently published study was approximately 35% within 72 hours of ED discharge.[50] We can therefore reasonably assume this proportion may approach 50% by day 7. Using the 50% estimate is conservative since it requires the largest sample size, providing at least 80% power regardless of the observed proportions. This is a superiority study in which the adoption of nasal suctioning will be recommended for future practice if the observed proportion of the primary outcome in this group is significantly lower than in the controls. With 186 patients per arm (372 in total) a two-sided test with a type I error of 0.05 will have 80% power to achieve statistical significance if suctioning reduces the probability of treatment failure from 50% to 35% (i.e. absolute reduction of 15%). This estimate is based on clinically relevant differences agreed upon by study investigators and it also represents an NNT of 7. In the Cochrane review of asthma therapies an NNT of a comparable magnitude led to a change in national practice recommendations.[52] Since bronchiolitis and related medical visits are highly prevalent,[20] this target difference would also have an important economic impact. Based on our previous bronchiolitis trials, the anticipated refusal rate may be 20%. Given the study design and our past experience, the study non-completion rate and loss to follow-up can safely be assumed to be no higher than 5% each. Therefore, to have complete data on 372 patients we plan to randomize 412 (*i.e.* 372/ (1 - 0.05) *(1 – 0.05) and to approach 515 (*i.e.* 412/(1 - 0.20).

Feasibility

We plan to implement an enrollment schedule similar to the one used in previous similar studies, for a total of 72 hours a week. Extensive weekly coverage is needed since the time of presentation of these children varies. These hours will be covered by several trained on call research nurse/research assistants. Current estimates point to annual ED volumes at SickKids, CHEO, Children's Hospital of Western Ontario London and McMaster Children's Hospital of 180,000 patients total, approximately 1600 of who have bronchiolitis each winter. Based on our recent bronchiolitis study at SickKids, we expect 800 (50%) will be screened each season,

480 will be discharged home (40% admission rate), 288 (60%) will meet other eligibility criteria and 230 will be randomized each season (20% refusal rate). Assuming a 5% study non-completion rate and a 5% loss to follow up rate, 206 patients with a full set of data can be expected at all 4 participating EDs each winter. Therefore, 2 bronchiolitis winter seasons represent an adequate timeframe to accrue the needed sample size. Since virtually all bronchiolitis cases occur between December and March, enrollment will be limited to these periods.

Compliance issues

In this study it is important to achieve a balance between adhering to the experimental intervention and to simulate a real-life practice. Since this is a pragmatic trial, no reminders after ED discharge will be implemented. We have also adjusted the sample size to account for the possibility of study non-completion.

We expect that compliance will be enhanced by our provision of the suctioning device to the interventional group and by implementing parental counselling about the association between nasal obstruction and feeding, breathing and sleeping challenges. This real world, pragmatic effectiveness design is an important consideration in clinical trials.[53,54,55] We will measure compliance during the telephone/e-mail follow up by asking caretakers about the kind of suctioning device used and the frequency of use. We will also conduct an exploratory "as-treated" analysis to determine if those who were compliant with intervention had superior outcomes. The questionnaire also targets the follow up question asking the families if they have used the suctioning device prior to feeds most of the time.

Analyses

The principle of intent-to-treat will be applied to all analyses.

The primary analysis will be a two-sided Fisher's Exact test to test the null hypothesis that there is no difference in the treatment arms with respect to the probability of treatment failure. This analysis will be performed using a two-sided test of hypothesis with a type I error of 0.05.

Since this trial involves a minimal risk to study subjects, an interim analysis will not be performed.

Secondary analyses:

All secondary analyses will be 2-sided and use a Bonferroni correction (0.05/6=0.0083) for the level to declare significance.

1- The Fisher's Exact test will be used to compare the proportions of parents in the two study groups reporting unscheduled medical visits for bronchiolitis within 7 days of discharge from the index ED visit.
2- The Fisher's Exact test will be used to compare the proportions of parents reporting ED visits for bronchiolitis within 7 days of discharge from the index ED visit.
3- The Fisher's Exact test will be used to compare the proportions of parents reporting normal/almost normal feeding pattern on day 7.
4- The Fisher's Exact test will be used to compare the proportions of parents reporting normal/almost normal sleeping of their child on day 7.
5- The Fisher's Exact test will be used to compare the proportions of parents reporting their own normal/almost normal sleeping on day 7.
6- The Fisher's Exact test will be used to compare the proportions of parents reporting their perceived level of satisfaction with care for their child's illness after discharge from the ED as either "very satisfied" or "satisfied" on day 7.

As a secondary analysis we will also test for heterogeneity of treatment effect between sites and, if required, apply a random-effects model for comparing treatment groups across sites.

Day to Day Management of the Study

Prior to the study, the co-investigators will educate all ED physicians and nurses/research assistants at the participating sites re the details of the study, hire and train the research staff in the best practice guidelines, accurate data entry and maintaining the study log. The study coordinator will be trained in the various administrative study aspects and is skilled in coordinating multi-site trials. She/he will report to the principal investigator and assist with logistics of the study, organize inter-site communications and check the incoming data for accuracy and completeness. The co-investigators at all centers will act as a local resource to the clinical research nurse/research assistants and the study managers. We plan several teleconference between collaborators to discuss any outstanding issues. Diane Soares, the collaborating respiratory therapist at SickKids will prepare and distribute the study kits to all study centers.

Roles of Applicants:

Dr. Schuh will be responsible for the overall coordination of the study. Drs. Finkelstein, Parkin, Zemek, Plint, Poonai and Eltorki will also be assisting Dr. Schuh. Dr. Coates will also act as respiratory consultant. Dr. Coates has also played a crucial role in piloting the aspiration device. Dr Willan will supervise the data analysis.

Trial Steering: Dr Schuh has published numerous single and multi-centre peer-reviewed and competitively funded RCTs of various interventions in asthma, bronchiolitis and croup in the ED. Dr. Schuh will, together with Dr. Plint, a co-investigator and a site PI on the current trial and an experienced CIHR-funded ED scientist, will coordinate the overall study. Dr. Plint has led a multi-site trial of bronchiolitis funded by the CIHR. Dr. Coates has special expertise in nebulization delivery of drugs. Dr Finkelstein has an extensive track record in successful publication of competitive peer-reviewed ED trials; Dr Zemek and Dr Parkin have published numerous pivotal studies enhancing the standard of practice of pediatric acute asthma and Dr Poonai also researches optimal management of common pediatric ailments in the ED. Dr. Willan has an international reputation in the design and analysis of randomized controlled trials.

Safety

Millions of parents worldwide use some form of nasal suctioning in bronchiolitis and this intervention is considered to be safe. Current literature also does not provide evidence of any side effects attributable to the superficial method of suctioning not involving the nasopharynx which will be used in this study. Side effects which may foreseeably happen with this method of suctioning are not likely to be serious and may include irritation of the nasal mucosa, epistaxis, irritability/crying and vomiting. Prolonged crying during suctioning lasting more than about 5 minutes will be tracked in all infants receiving any method of suctioning.

While theoretical side-effects of deep nasal suctioning also include bradycardia, laryngospasm and bronchospasm,[56] we shall not use this invasive sectioning method in this study. There is controversial retrospective inpatient evidence that deep suctioning may result in prolonged length of hospital stay in admitted infants[40,57] due to irritation of the upper airway with resultant edema. In this study we shall utilize a superficial, non-invasive suctioning method not anticipated to result in adverse events.

Adverse Event (AE): An adverse event is any unfavorable or unintended clinical or other occurrence during the study period that may or may not be the result of participation in the research study.

Expected Adverse Events

These include the following as they are part of the natural history of the underlying disease process, such as hospitalization, future health care provider visit, ED return visit, IV rehydration, supplemental oxygen therapy during subsequent visits, cough, nasal congestion, respiratory distress, fever, crying post suctioning, irritation to the nasal mucosa, vomiting. Since expected adverse events are part of the natural history of bronchiolitis, they will not need to be reported as Adverse Events. This information will be recorded in normal study data collection processes.

Serious Adverse Events

Any Serious Adverse Event (SAE) that occurs after suctioning had commenced following ED discharge will be reported to the Research Ethics Board (REB) of all institutions and to the Principal Investigator within 48 hours and the study subject will be followed until the conclusion of the event.

A SAE is defined as: death, life-threatening event in which the patient was at immediate risk of death; results in a disability or is medically significant. Important medical events may be considered SAEs when, based upon appropriate medical judgment, may require medical or surgical intervention to prevent one of the outcomes listed in this definition.

Adverse Event Reports

For unexpected adverse events, the site PI will inform their REB as per local guidelines.

Knowledge Translation Strategies

Current evidence-based management of bronchiolitis includes exclusive use of supportive care strategies. Providing the evidence of benefit of the most frequently used such strategy, nasal suctioning, will supply a critical piece of the puzzle in the effective symptomatic management of this common disease with a high nasal obstruction-related morbidity. The results of this trial will help guide optimal bronchiolitis management by health care providers and families and may significantly impact patient-care and healthcare costs.

Future dissemination of the results will include knowledge translation (KT), incorporation of the findings into national and international clinical practice guidelines for management of bronchiolitis as well as into a Cochrane review, presentation at scientific meetings and a peer-reviewed publication. We will also carry out presentations locally, present findings nationally through webinars and meetings of the Canadian Paediatric Society, Canadian Association of Emergency Physicians, and Society of Pediatric Nurses/research assistants and internationally at the Pediatric Academic Societies, American Academy of Pediatrics and the American College of Emergency Physicians.

As active members of national and international expert networks, we have a unique and powerful infrastructure to complete KT of the study results. Integrated KT employing pre-established collaborations with end-knowledge users [Pediatric Emergency Research Canada (PERC), Pediatric Emergency Research Network (PERN), Translating Emergency Knowledge in Kids (TREKK), Canadian Paediatric Society, American Academy of Pediatrics, Canadian Association of Emergency Physicians, American College of Emergency Physicians, Canadian Thoracic Society and American Thoracic Society] will result in broad dissemination to health care providers and the public. Critically, these organizations will incorporate this new evidence into their continuing education aimed at the primary care providers where many families get counselling on bronchiolitis. Our yearly Pediatric Emergency Conference attended by many primary care providers will further assist in achieving this goal. Using the Twitter, study evidence will also be disseminated to parenting groups, literature on health information for parents. We shall also educate Telehealth where many parents seek health care advice.

We will use PERC, TREKK and PERN to enhance front-line use of our results across Canada and beyond. Specifically, PERC network is part of the world-wide Pediatric Emergency Research Network (PERN), consisting of 122 hospitals on 5 continents. Recognizing that the majority of Canadian children receive emergency care in general EDs, knowledge mobilization is required from the academic pediatric emergency centers to the community. TREKK, funded by the Networks of Centers of Excellence Knowledge Mobilization initiative, aims to accelerate the speed at which the latest knowledge in children's emergency care is put into practice in general EDs. TREKK's knowledge mobilization initiative is grounded in the partnership and exchange between over 30 general EDs across Canada and members of PERC, PERN and KT Canada. Our end-of-grant KT through TREKK will facilitate bridging the research-to-practice gap and raise the overall standard of care for children visiting general EDs with bronchiolitis. Partnership with the Canadian Medical Association and the aforementioned professional organizations ensures that our findings will be employed by health care providers.

We look forward to the time when the emergency physicians and primary care practitioners incorporate suctioning guidelines into discharge instruction materials for their patients with

bronchiolitis. We are confident that the proposed study will lead to a higher standard of bronchiolitis care.

References

1. Ralston SL, Lieberthal AS, Meissner HC, et al. Clinical practice guideline: the diagnosis, management, and prevention of bronchiolitis. Pediatrics 2014;134:e1474-502.
2. Shay DK, Holman RC, Newman RD, Liu LL, Stout JW, Anderson LJ. Bronchiolitis-associated hospitalizations among US children, 1980-1996. Jama 1999;282:1440-6.
3. Pelletier AJ, Mansbach JM, Camargo CA, Jr. Direct medical costs of bronchiolitis hospitalizations in the United States. Pediatrics 2006;118:2418-23.
4. Hall CB, Weinberg GA, Iwane MK, et al. The burden of respiratory syncytial virus infection in young children. N Engl J Med 2009;360:588-98.
5. Hasegawa K, Tsugawa Y, Brown DF, Mansbach JM, Camargo CA, Jr. Trends in bronchiolitis hospitalizations in the United States, 2000-2009. Pediatrics 2013;132:28-36.
6. Ehlken B, Ihorst G, Lippert B, et al. Economic impact of community-acquired and nosocomial lower respiratory tract infections in young children in Germany. Eur J Pediatr 2005;164:607-15.
7. Yorita KL, Holman RC, Sejvar JJ, Steiner CA, Schonberger LB. Infectious disease hospitalizations among infants in the United States. Pediatrics 2008;121:244-52.
8. Mansbach JM, Emond JA, Camargo CA, Jr. Bronchiolitis in US emergency departments 1992 to 2000: epidemiology and practice variation. Pediatric Emergency Care 2005;21:242-7.
9. Norwood A, Mansbach JM, Clark S, Waseem M, Camargo CA, Jr. Prospective multicenter study of bronchiolitis: predictors of an unscheduled visit after discharge from the emergency department. Acad Emerg Med 2010;17:376-82.
10. Reynolds EO, Cook CD. The Treatment of Bronchiolitis. J Pediatr 1963;63:1205-7.
11. American Academy of Pediatrics Subcommittee on D, Management of B. Diagnosis and management of bronchiolitis. Pediatrics 2006;118:1774-93.
12. Friedman JN, Rieder MJ, Walton JM, Canadian Paediatric Society ACCDT, Hazardous Substances C. Bronchiolitis: Recommendations for diagnosis, monitoring and management of children one to 24 months of age. Paediatr Child Health 2014;19:485-98.
13. Network SIG. Bronchiolitis in Children: A national clinical guideline2006.
14. Turner T, Wilkinson F, Harris C, Mazza D, Health for Kids Guideline Development G. Evidence based guideline for the management of bronchiolitis. Aust Fam Physician 2008;37:6-13.

15. Barben J, Kuehni CE, Trachsel D, Hammer J, Swiss Paediatric Respiratory Research G. Management of acute bronchiolitis: can evidence based guidelines alter clinical practice? Thorax 2008;63:1103-9.
16. Excellence NIfHaC. Bronchiolitis in children: Diagnosis and management. UK2015.
17. Florin TA, Byczkowski T, Ruddy RM, Zorc JJ, Test M, Shah SS. Variation in the management of infants hospitalized for bronchiolitis persists after the 2006 American Academy of Pediatrics bronchiolitis guidelines. J Pediatr 2014;165:786-92 e1.
18. Christakis DA, Cowan CA, Garrison MM, Molteni R, Marcuse E, Zerr DM. Variation in inpatient diagnostic testing and management of bronchiolitis. Pediatrics 2005;115:878-84.
19. Plint AC, Johnson DW, Wiebe N, et al. Practice variation among pediatric emergency departments in the treatment of bronchiolitis. Acad Emerg Med 2004;11:353-60.
20. Mansbach JM, Emond JA, Camargo CA, Jr. Bronchiolitis in US emergency departments 1992 to 2000: epidemiology and practice variation. Pediatric emergency care 2005;21:242-7.
21. Cheung CR, Smith H, Thurland K, Duncan H, Semple MG. Population variation in admission rates and duration of inpatient stay for bronchiolitis in England. Archives of disease in childhood 2013;98:57-9.
22. Knapp JF, Simon SD, Sharma V. Quality of care for common pediatric respiratory illnesses in United States emergency departments: analysis of 2005 National Hospital Ambulatory Medical Care Survey Data. Pediatrics 2008;122:1165-70.
23. Knapp JF, Hall M, Sharma V. Benchmarks for the emergency department care of children with asthma, bronchiolitis, and croup. Pediatric emergency care 2010;26:364-9.
24. Plint AC, Grenon R, Klassen TP, Johnson DW. Bronchodilator and steroid use for the management of bronchiolitis in Canadian pediatric emergency departments. CJEM 2015;17:46-53.
25. Ralston S, Comick A, Nichols E, Parker D, Lanter P. Effectiveness of quality improvement in hospitalization for bronchiolitis: a systematic review. Pediatrics 2014;134:571-81.
26. Macias CG, Mansbach JM, Fisher ES, et al. Variability in inpatient management of children hospitalized with bronchiolitis. Acad Pediatr 2015;15:69-76.
27. Behrendt CE, Decker MD, Burch DJ, Watson PH. International variation in the management of infants hospitalized with respiratory syncytial virus. International RSV Study Group. Eur J Pediatr 1998;157:215-20.
28. Chirico G, Beccagutti F. Nasal obstruction in neonates and infants. Minerva Pediatr 2010;62:499-505.
29. Walsh S. Infant with feeding difficulties. J Pediatr Health Care 2002;16:204, 9-10.
30. Tamura S, Kurata T. Defense mechanisms against influenza virus infection in the respiratory tract mucosa. Jpn J Infect Dis 2004;57:236-47.
31. Zuercher AW, Coffin SE, Thurnheer MC, Fundova P, Cebra JJ. Nasal-associated lymphoid tissue is a mucosal inductive site for virus-specific humoral and cellular immune responses. J Immunol 2002;168:1796-803.
32. Mallory MD, Shay DK, Garrett J, Bordley WC. Bronchiolitis management preferences and the influence of pulse oximetry and respiratory rate on the decision to admit. Pediatrics 2003;111:e45-51.
33. Baraldi E, Lanari M, Manzoni P, et al. Inter-society consensus document on treatment and prevention of bronchiolitis in newborns and infants. Ital J Pediatr 2014;40:65.
34. Bronchiolitis WGotCPGoA. Clinical Practice Guidelines in the Spanish Healthcare System2010.
35. Verstraete M, Cros P, Gouin M, et al. [Update on the management of acute viral bronchiolitis: proposed guidelines of Grand Ouest University Hospitals]. Arch Pediatr 2014;21:53-62.
36. Oymar K, Skjerven HO, Mikalsen IB. Acute bronchiolitis in infants, a review. Scand J Trauma Resusc Emerg Med 2014;22:23.

37. Nagakumar P, Doull I. Current therapy for bronchiolitis. Arch Dis Child 2012;97:827-30.
38. Moschino L, Mario F, Carraro S, Visentin F, Zanconato S, Baraldi E. Is nasal suctioning warranted before measuring O2 saturation in infants with bronchiolitis? Arch Dis Child 2016;101:114-5.
39. Krugman SD, Bhagtani HR. Parental perception of the effectiveness of treatments for infant nasal congestion. Clin Pediatr (Phila) 2013;52:762-4.
40. Mussman GM, Parker MW, Statile A, Sucharew H, Brady PW. Suctioning and length of stay in infants hospitalized with bronchiolitis. JAMA Pediatr 2013;167:414-21.
41. Zorc JJ, Hall CB. Bronchiolitis: recent evidence on diagnosis and management. Pediatrics 2010;125:342-9.
42. Ford I, Norrie J. Pragmatic Trials. N Engl J Med 2016;375:454-63.
43. Voets S, van Berlaer G, Hachimi-Idrissi S. Clinical predictors of the severity of bronchiolitis. Eur J Emerg Med 2006;13:134-8.
44. Ducharme FM, Dell SD, Radhakrishnan D, et al. Diagnosis and management of asthma in preschoolers: A Canadian Thoracic Society and Canadian Paediatric Society position paper. Paediatr Child Health 2015;20:353-71.
45. Moher D, Hopewell S, Schulz KF, et al. CONSORT 2010 explanation and elaboration: updated guidelines for reporting parallel group randomised trials. BMJ 2010;340:c869.
46. Lewis JA, Machin D. Intention to treat--who should use ITT? Br J Cancer 1993;68:647-50.
47. Thompson M, Vodicka TA, Blair PS, et al. Duration of symptoms of respiratory tract infections in children: systematic review. BMJ 2013;347:f7027.
48. Schuh S, Freedman S, Coates A, et al. Effect of oximetry on hospitalization in bronchiolitis: a randomized clinical trial. Jama 2014;312:712-8.
49. Pruikkonen H, Uhari M, Dunder T, Pokka T, Renko M. Infants under 6 months with bronchiolitis are most likely to need major medical interventions in the 5 days after onset. Acta Paediatr 2014;103:1089-93.
50. Principi T, Coates AL, Parkin PC, Stephens D, DaSilva Z, Schuh S. Effect of Oxygen Desaturations on Subsequent Medical Visits in Infants Discharged From the Emergency Department With Bronchiolitis. JAMA Pediatr 2016;170:602-8.
51. Petruzella FD, Gorelick MH. Duration of illness in infants with bronchiolitis evaluated in the emergency department. Pediatrics 2010;126:285-90.
52. Rowe BH, Spooner C, Ducharme FM, Bretzlaff JA, Bota GW. Early emergency department treatment of acute asthma with systemic corticosteroids. Cochrane Database Syst Rev 2001:CD002178.
53. Maclure M. Explaining pragmatic trials to pragmatic policy-makers. CMAJ 2009;180:1001-3.
54. Zwarenstein M, Treweek S. What kind of randomized trials do we need? CMAJ 2009;180:998-1000.
55. Thorpe KE, Zwarenstein M, Oxman AD, et al. A pragmatic-explanatory continuum indicator summary (PRECIS): a tool to help trial designers. CMAJ 2009;180:E47-57.
56. Czarnecki ML, Kaucic CL. Infant nasal-pharyngeal suctioning: is it beneficial? Pediatr Nurs 1999;25:193-6, 218.
57. Weisgerber MC, Lye PS, Li SH, et al. Factors predicting prolonged hospital stay for infants with bronchiolitis. J Hosp Med 2011;6:264-70.

AMENDED PROTOCOL

Suctioning of NOse Therapy (SNOT) in Bronchiolitis - a Randomized Clinical Trial

Principal Investigator:

Dr. Suzanne Schuh — The Hospital for Sick Children, Toronto, Ontario

Co-Principal Investigator:

Dr. Yaron Finkelstein — The Hospital for Sick Children, Toronto, Ontario

Co-Investigators:

Dr. Patricia Parkin — The Hospital for Sick Children, Toronto, Ontario

Dr. Allan Coates — The Hospital for Sick Children, Toronto, Ontario

Dr. Amy Plint — The Children's Hospital of Eastern Ontario, Ottawa, Ontario

Dr. Roger Zemek — The Children's Hospital of Eastern Ontario, Ottawa, Ontario

Dr. Naveen Poonai — London Health Sciences Centre, London, Ontario

Dr. Mohamed Eltorki — McMaster Children's Hospital (Hamilton Health Sciences), Hamilton, Ontario

Collaborator:

Diane Soares RRT — The Hospital for Sick Children, Toronto, Ontario

BACKGROUND

Burden of bronchiolitis: Bronchiolitis is a viral syndrome of infants under 12 months of age, characterized by the first episode of rhinorrhea, cough and respiratory distress[1]. Bronchiolitis represents a disease with low mortality rates but very high morbidity; it is the leading cause of infant hospitalizations in the Western world, with increasing costs over the past several decades.[1-3] In the United States, bronchiolitis-related annual hospitalization expenses exceed $500 million,[4,5] with similar high economic burden in other Western countries.[6] More than one third of all infants develop bronchiolitis within the first year of life, of whom 3% are admitted to hospital.[7] Of infants diagnosed with bronchiolitis in the Emergency Departments (ED), 30-40% requires hospitalization.[8] The reported annual healthcare utilization rate for bronchiolitis per 1000 infants under 6 months of age is 17 hospitalizations, 55 ED visits, and 132 unplanned office visits.[3] Return visits are also commonplace: a large prospective multicenter study demonstrated that 20% of infants discharged home with bronchiolitis from the Emergency Departments (ED) in the United States have unscheduled visits for persistent or worsening symptoms within the subsequent 2 weeks.[9] Therefore, bronchiolitis is an extremely common and expensive problem, with considerable impact on families!

Challenges with management of bronchiolitis: Despite extensive research, current principles for bronchiolitis management remain similar to those recommended half a century ago.[10] Since effective therapies for bronchiolitis remain elusive, the American Academy of Pediatrics (AAP), Canadian Paediatric Society and other professional societies advocate for the use of supportive measures such as adequate oxygenation, hydration and airway stabilization and discourage routine use of pharmacotherapy.[1,11-16] The paucity of effective evidence-based therapy has led to substantial practice variation, including use of unwarranted treatments.[17-26] Indeed, management of bronchiolitis is more strongly associated with local hospital practices or individual physician preferences than with disease severity[27].

Since guidelines recommend offering only supportive care to infants with bronchiolitis, it is important to determine which aspects of supportive care are effective. While little doubt exists about the need for intravenous rehydration in lethargic, dehydrated infants and for supplemental oxygen in infants with respiratory distress and significant hypoxemia, no previous study has examined the benefits of nasal suctioning in bronchiolitis.

Importance of Nasal Patency in Bronchiolitis: An important challenge associated with caring for patients with bronchiolitis concerns the fact that it involves young infants less than one year of age. Young infants are obligate nose breathers[28] and their inability to remove nasal secretions by nose blowing contributes to bronchiolitis morbidity such as augmented respiratory distress, altered sleep cycle and feeding difficulties.[28,29] Maintaining hydration is critically important in bronchiolitis care: poor feeding associated with respiratory distress represents a major reason for admitting babies with bronchiolitis to hospital.[28]

Although bronchiolitis involves primarily the lower respiratory tract, dysfunction of the upper airway also plays a key role in the pathogenesis, with nasal edema and inflammatory exudate as its integral components. Furthermore, as much as 50% of the total airway resistance originates in the nose which plays a key role in humidification of inhaled air, ciliary activity as well as in providing innate immunity and modulation of inflammation.[28,30,31]

What is known about nasal suctioning in bronchiolitis? Although there is no proven therapy to ameliorate the lower airway pathology in bronchiolitis, many practitioners employ supportive measures related to relieving nasal obstruction. Specifically, a study by Mallory which examined common treatment strategies in bronchiolitis employed by 519 pediatricians in the United States reported that 82% of the responders utilized nasal suctioning as the most common therapeutic strategy and this proportion exceeded the frequency of all other treatment approaches.[32] The authors emphasize that *this commonly used intervention is not evidence-based and needs to be further explored.*[32]

Based on intuitive practice rather than on proven evidence, several comprehensive bronchiolitis guidelines from North America, Europe and Australia as well as some reviews

recommend consideration of nasal suctioning as a therapeutic option. These include the NICE guideline from the U.K., the Canadian Pediatric Society Guideline, the Scottish Intercollegiate Guidelines Network (SIGN), the inter-society consensus document from Italy, Clinical Practice Guidelines in the Spanish National Healthcare System, and the HUGO guideline from France.[12-14,33-37] The SIGN and American Academy of Pediatrics (AAP) 2014 guidelines highlight the expert consensus as the basis for this recommendation, and point out lack of relevant evidence supporting its benefit.[1,13] Literature also suggests plausible but unproven hypotheses that the transcutaneous oxygen saturation should be measured after nasal aspiration.[28,38] A recent parental survey found that 90% of parents perceive nasal suctioning as effective in improving infants' symptoms.[39] Given how frequently suctioning is employed, *the benefits of this commonly employed intervention need to be established before this treatment modality can be recommended.* Furthermore, although the widespread superficial method to be used in this study of nasal suctioning is generally regarded to be safe, the side-effects of this intervention have not been systematically documented. Some healthcare providers find that infants sometimes perceive nasal suctioning as unpleasant (see also Safety section) and we shall thus track adverse effects of suctioning during the study. Therefore, even if the study yields negative results, this would provide important evidence and inform clinical care.

Suctioning device:

In a study of nasal suctioning, it is imperative to implement an effective suctioning tool. We have investigated several such devices available on the Canadian market. The main disadvantage of the oral suctioning units (where the caretaker suctions the nose) such as Hydrasense (Shering Plough Canada), Naspira (NeilMed Pharmaceuticals) and Nosefrida is their operator effort- dependence and consequent variability in the generated negative pressures and aspirated volumes. Furthermore, we found that the aspirated fluid consistently crossed the filter barrier, posing a potential infection safety issue to the operator. The ubiquitous bulb aspirators are limited by a very short time interval of ≤ 1 second available for bulb release during aspiration which results in non-sustained aspiration pressures. Therefore, the use of the bulb device would result in a non-sustained suctioning effect. The Babyvac device (Illes Csok Es

TSA, Budapest, Hungary) has to be attached to a vacuum cleaner which may prevent many parents from using it. This device is also noisy and generates variable pressures, depending on the vacuum model.

In contrast, the battery-operated device by Zo-Li (Zo-Li, Inc, CA, U.S.A.) generates consistent and reproducible high negative pressure of 126 mm Hg which is sustained over the 10 second suctioning interval. This pressure is comparable to the 120 mm Hg pressure which represents the accepted Canada-wide hospital standard for the wall suction available in the EDs and other hospital settings. The interface is a nasal cushion that makes a tight but comfortable seal at the nares. An important advantage of the Zo-Li device over other available suctioning tools is that is operator effort- independent and has consistent performance.

Recent study pilot: we have just carried out a pilot phase of 35 infants randomized to either suctioning via the ZoLi device versus usual care for 7 days, with no clinical difference in the proportion of subsequent visits. In the pilot we have learned that virtually all parents already suction or expect to suction their child's nose with one of the devices available on the market, and the expected difference in benefit between the study groups would thus be minimal. To assess the benefit of suctioning, the control group therefore needs to undergo either no suctioning or suctioning with minimal effect. In view of the widespread use of suctioning, use of no intervention in the controls would likely meet with parental resistance and enrollment would be difficult. Therefore, we have opted for the latter option.

Prior to the pilot, we have investigated various suctioning devices. We have found that the ubiquitous bulb aspirators are limited by a very short time interval of ≤ 1 second available for bulb release during aspiration which results in non-sustained aspiration pressures and minimal aspirated volumes. Therefore, the bulb suction would provide a very mild suctioning effect and would serve as a reasonable placebo equivalent. Since the benefit of nasal suction in bronchiolitis is currently unknown, this design is ethically acceptable. During the pilot we have also learned that the majority of parents stop suctioning within 3-4 days, and prolonged study duration is therefore unlikely to be fruitful. Since the vast majority of infants with bronchiolitis

seen in the ED who re-present do so within 72 hours, we shall choose this time interval for outcome determination.

Research Questions:

<u>Primary:</u>

In otherwise healthy infants 4 weeks to 12 months of age discharged home from the ED with acute bronchiolitis,[1] is there a difference in the probability of “treatment failure” by 72 hours post discharge from the index ED visit in those receiving nasal suctioning via Zo-Li device prior to feeds compared to infants who receive nasal suctioning via bulb? Treatment failure is defined as 1) any bronchiolitis-related healthcare visit, except visits that have occurred only due to ED-recommendation at time of ED discharge or 2) the use of additional (non-study assigned) suctioning devices (see Outcome Measures section) within approximately 72 hours post discharge at the index ED visit.

<u>Hypothesis:</u>

We hypothesize that the infants who undergo scheduled nasal suctioning via ZoLi device will experience a lower treatment failure probability by approximately 72 hours post discharge from index ED visit compared to those managed by suction via a bulb.

<u>Secondary:</u>

7- In these infants, is there a difference in the mean number of medical visits for bronchiolitis (defined as #1 under primary outcome above) within 72 hours of ED discharge?
8- In these infants, is there a difference in the mean number of unscheduled medical visits for bronchiolitis within 72 hours of ED discharge?
9- In these infants, is there a difference in the mean number of ED visits for bronchiolitis within 72 hours of ED discharge?
10- In these infants, is there a difference in the probability of a parent reporting normal/near normal feeds on approximately 72 hours discharge?

11- In these infants, is there a difference in the probability of a parent reporting normal/near normal sleeping on approximately 72 hours post ED discharge?
12- For the parent, is there a difference in the probability of a parent reporting their own normal/near normal sleeping on approximately 72 hours post ED discharge?
13- In these infants, is there a difference in the probability of parents reporting at 72 hours post ED discharge as being "very satisfied" or "satisfied" with their ability to care for their child during the study period?

Study Design:

This is a multi-center, randomized, outcome assessor- blinded clinical trial of infants discharged home from the ED with acute bronchiolitis. Two groups will be compared: infants receiving bronchiolitis suctioning via a bulb (see below) versus those given nasal suctioning via a battery-operated nasal aspiration Zo-Li device just prior to each feeding for 72 hours post index ED visit discharge. Evidence suggests that suctioning at frequent intervals is associated with movement of secretions from the lower airway with improved minute volume.[40] The 72 hour study period has been chosen since the majority of infants discharged from the ED with bronchiolitis experience the targeted outcomes within this time frame.[9]

Prior to the ED discharge, all study participants will receive usual bronchiolitis care ordered by the ED physician as per standard of care which consists of supplemental oxygen for saturations <90% and supplemental IV hydration in those with inadequate oral intake.[41] Eligible consenting patients deemed suitable for discharge home (representing approximately 60-70% of the bronchiolitis population presenting to the ED) will be randomly allocated to one of two study groups:

<u>Control Group (Group 1):</u> this group will receive standardized routine discharge instructions describing information about bronchiolitis, expected course of illness, recommended management strategies such as fever control, augmented air humidification, need for frequent feeding and warning signs prompting return for care. This group will be suctioned prior to each feeding as needed for 72 hours post discharge home via bulb suction (with saline drops) which

is expected to provide minimal effect, due to non-sustained negative pressures generated during bulb release. Since the benefit of nasal suction in bronchiolitis is unknown, this design is ethically reasonable. However, the use of no suction would likely meet with parental resistance and enrollment would be difficult. Families in the control group will be given the bulb device at no cost and instructed in the appropriate technique of using this tool prior to feeds.

Interventional Group (Group 2): in addition to receiving the aforementioned bronchiolitis discharge instructions, this group will undergo nasal suctioning prior to each feeding as needed for 72 hours post discharge home, using exclusively the Zo-Li study device (see above under study device), with saline nose drops. Families in this group will be given the Zo-Li device at no cost and instructed in the appropriate technique and importance of using this tool.

We shall not reveal the identity of the study devices to the ED physicians in order to minimize contamination of the control group. The ED treating physicians will also be blinded to which device the infant had been randomized to. We shall also ask the ED treating physicians not to recommend specific suctioning devices to the study patients.

Study Population:

Inclusion criteria:

6- Diagnosis of bronchiolitis in the ED as per the AAP diagnosis definition. [1]Bronchiolitis is defined as the first episode of viral upper respiratory infection with respiratory distress and/or tachypnea for age.
7- Age 4 weeks up to and including 12 months of age. Participating infants will have to be at least 4 weeks post their expected due date of birth since infants with bronchiolitis below this age cut-off are at a much higher risk of apnea and dehydration than their older counterparts.[42]
8- Nasal congestion as per parental report and/or the treating physician
9- Must have at least one of the following: home/cellular telephone or e- mail
10- Informed consent.
11- Parent/caregiver speaks English/French.

Exclusion criteria:

7. Previous diagnosis of bronchiolitis made more than 3 weeks prior to this ED visit.[43]
8. Hospitalization at the index ED visit. Although we have considered starting the experimental intervention at presentation to the ED, this was deemed counterproductive since many patients with bronchiolitis are currently routinely suctioned in the ED which would likely contaminate the control arm and impact study results.
9. Use of any battery operated suctioning device prior to arrival. These families may choose to continue using these electrical devices which would contaminate the study groups.
10. Co-morbidities which may impact outcomes such as known diagnosis of congenital heart disease, chronic respiratory disease including known lung disease due to prematurity, aspiration due to severe gastro-esophageal reflux, neuro-muscular/neurologic disease, immunodeficiency, coagulopathies, nasal/upper airway abnormalities, oral, gastrointestinal anomalies [except for corrected pyloric stenosis], tracheo-esophageal fistulas, gastric/gastro-jejunal tube feeding supplementation.

Sample selection:

Infants presenting to the collaborating EDs who meet eligibility criteria will be approached for enrollment when the research nurse/research assistants are on duty (days and evenings). The research nurse/research assistants will keep a log of all infants presenting to the ED with bronchiolitis during the study period whether randomized or not in order to confirm lack of enrollment bias. Specifically, information collected in non-enrolled patients will include age, gender and disposition from the ED. All of the collaborating hospitals are paediatric tertiary care centers which see the entire clinical and demographic spectrum of the bronchiolitis population. Our profile of children with bronchiolitis is therefore comparable to that of other institutions, the generalizability of the study should not be affected and referral bias should be minimal. A standardized data collection form will be used to assess the baseline and demographic features that may affect outcome and potentially confound the comparisons.

Since the patients will be screened consecutively and study coverage will occur during days, evenings and weekends, selection bias should not play a significant role.

Allocation to Study Groups:

Sequence generation: Data management department at the Research Institute at the Hospital for Sick Children will provide data management services for this study. In this study, we shall use a third-party treatment allocator, the randomization service www.randomize.net for treatment assignment. We have successfully used this service in previous trials. This service will produce and securely guard a randomization list produced with the help of random-number generating software. *Allocation concealment*: This randomization service will allocate eligible consenting patients to the two study groups, with the assignment stratified by site and age (< 6 months vs ≥ 6 months), using a permuted block randomization of 4 and 6 with a 1:1 allocation ratio. The allocation sequence will be securely stored at the randomization service site until study enrollment has been concluded and the analyses have been finalized. *Implementation:* Upon receiving the e- mail indicating the study number and group assignment of a given participant, the study nurse will obtain the next consecutively numbered study kit for that patient and enter the number into a confidential log book. The kits will contain standardized bronchiolitis discharge instructions which will be supplemented with the Zo-Li suctioning device or the bulb device and written instructions on their use. The study kits, the Zo-Li interventional device, the control bulb device and the logbook will be kept in a locked research cabinet in the ED.

Minimizing bias:

Bias will be minimized by strictly adhering to the 2010 CONSORT Statement recommendations including the use of "third-party" treatment assignment.[44] For practical reasons, it is not feasible to blind parents to study intervention. However, the caregivers filling out the electronic follow up survey will be blinded to study hypothesis, as will be the research assistants performing telephone follow up and the data analyst: re the ascertainment bias will thus be minimized. The ED treating physicians will also be blinded to treatment assignment. To blind the study analyst

to treatment allocation, the assigned allocation in the electronic study database will be designated only by an unidentified “group 1” or “group 2” designation. Furthermore, the primary outcome (bronchiolitis-related medical visits and hospitalizations or use of non-assigned suctioning devices) is a health care resource utilization variable. An intention-to-treat analysis will be performed to minimize bias associated with poor compliance and non-random loss of participants.[45] Co-interventions (e.g. use of other suctioning devices) and other sources of confounding will be recorded. Reporting bias will be avoided by registering the study at clinicaltrials.gov.

Referral bias is unlikely since all participating EDs see patients with the entire spectrum of bronchiolitis severity and the results should be also generalizable to the general EDs. The patients will be screened consecutively and study coverage will occur days, evenings and weekends. The study nurse/research assistants will keep continuous study logs with the characteristics including age, gender and vital signs in triage of the participating and non-participating patients which will also track missed patients, those excluded for criteria and patients who refused participation to document any selection bias.

Pre-study screening and baseline evaluation

All previously healthy infants 4 weeks to 12 months of age with the first episode of respiratory distress and upper respiratory infection will have vital signs and oxygen saturation assessed by the triage staff, as per usual clinical routine and will undergo treatment as per local standard of bronchiolitis care.

The research nurse/research assistants on duty will pre-screen potentially eligible patients for age and for respiratory complaints by reviewing the Electronic Data Information System triage note and by communication with the treating physician about the confirmed diagnosis of bronchiolitis. Once permission to be approached for research has been obtained from the caretakers using site specific and privacy office approved approach methods, the research nurse/research assistants on duty will assess eligibility and obtain written informed consent

after planned discharge home has been communicated. Thereafter, the research nurse/research assistants will document relevant demographic and clinical information.

Study procedure:

4- The eligible consented participants will be randomly allocated by www.randomize.net to the two study groups. All participants will be given standardized bronchiolitis discharge instructions according to each individual site regarding anticipated duration of symptoms of up to 2-3 weeks,[46] need for small, frequent feeds and fever control, and encouragement to return to ED if there is marked decrease in current feeding pattern, inappropriate lethargy, poor urine output/no wet diapers in 12 hours, more labored/rapid breathing. Those assigned to the interventional group will be provided with the Zo-Li suctioning device and instructed on its use and care, as will be the families assigned to suction with a bulb (controls).

2- Prior to discharge home, the study nurse will measure the vital signs, neck/chest retractions and transcutaneous oxygen saturation as per usual routine, to document comparable disease severity in the groups. For this purpose, the research nurse will also ask the caretakers about their child's feeding and sleeping adequacy and their own sleeping adequacy over the past 24 hours by asking the following Likert scale questions: 1) a) normal/almost normal amount [more than about 80% normal bottle intake or no/minimal difficulty breastfeeding]), b) Below normal but more than ½ usual bottle intake or difficulty breastfeeding c) very much less than normal (less than ½ usual bottle intake or major difficulty breastfeeding[16]. 2) "In the past 24 hours, how has your child slept compared to usual?" a) very much less than normal, b) less than normal), c) normal/almost normal d) somewhat more than normal e) very much more than normal 3) "In the past 24 hours, how have <u>you (the caregiver)</u> slept compared to usual?" a) very much less than normal b) somewhat less than normal c) normal/almost normal d) somewhat more than normal e) very much more than normal

5- In addition to the routine standardized discharge instructions given to all study patients, the patients in both study groups will receive detailed instructions on the proper use of the Zo-Li device (interventional group) and the bulb (control group) and on the importance of frequent suctioning prior to all feeds for the entire 72-hour study period.

6- All study patients will be encouraged to contact the study team should they have any questions about the suctioning technique

7- A follow-up questionnaire will be automatically sent to the caregiver's email address via secure SickKids REDCap database at 72 hours. Should the family choose a telephone follow-up instead, these patients will be contacted at approximately 72 hours by a research assistant/nurse trained in interviewing techniques and blinded to the study hypothesis and intervention assignment to determine subsequent hospitalization, family and physician-initiated medical visits, the level of feeding and sleeping adequacy and the level of perceived parental satisfaction to care for their child's illness.

Also, the use of any other suctioning devices will be recorded, as will be information about the potential adverse effects due to suctioning such as nose bleeding, prolonged screaming >5 minutes after suctioning, vomiting, and increased respiratory distress. We shall also ask about parental satisfaction with the suctioning device (any) used by asking the following Likert scale questions: 1) "During the past week, I have used device "x" to suction my child's nose. The following best describes my satisfaction with this device: a) very satisfied, b) satisfied, c) neither satisfied nor dissatisfied, d) dissatisfied, e) very dissatisfied, f) not applicable- no suction tool used". The assistant will also ask the dissatisfied parents a qualitative question about what their reasons for dissatisfaction were such as associated noise from the device, inconvenience, excessive irritability of the infant, persistent nasal congestion after the use of the device and the side effects listed above: 2) "Please tell us about your agreement with using the same suctioning tool again": a) very much agree, b) agree, c) neither agree not disagree, d) disagree,

e) very much disagree, f) not applicable. These responses will be used in a descriptive rather than analytical manner.

Outcome Measures

The primary outcome measure will be a composite outcome of "treatment failure" due to bronchiolitis symptoms, defined as: a) any bronchiolitis-related healthcare visit within 72 hours post discharge from the index ED visit, except visits that have occurred only due to the ED-recommendation at time of ED discharge (and not due to concern about bronchiolitis symptoms) or b) use of suctioning devices other than the one the participant has been assigned to use, due to parental or physician concern about poor feeding or respiratory distress. The visits counted as treatment failure will include 1) re-visits resulting in hospitalization for bronchiolitis to any inpatient facility or 2) family-initiated (unscheduled) bronchiolitis-related medical visits with any health care provider or 3) physician-initiated visits for bronchiolitis (such as the re-visits suggested by the primary care provider). All components of this outcome reflect concern about respiratory distress or inadequate feeding. In our past bronchiolitis study, we have determined that a considerable portion of infants with bronchiolitis experience subsequent medical visits due to persistent feeding/respiratory symptoms.[47]

In the pilot phase, we found that virtually all parents use some form of nasal suctioning. Because the minimal suctioning achieved with the use of the bulb may be perceived to be associated with feeding problems/respiratory discomfort, some parents may choose to use additional suctioning devices during the study period. This co-intervention thus also represents treatment failure: it will be tracked and has been incorporated into the outcome.

Bronchiolitis symptoms are most severe and impactful during the first week of illness,[48] about a third of infants with bronchiolitis discharged from the EDs have subsequent bronchiolitis-related visits[49] and the overwhelming majority of these occur within 72 hours of the initial presentation.[9] During the pilot phase we have learned that many parents stop suctioning after 3-4 days. Also, in view of the minimal suctioning effect of the bulb, some parents may choose

to buy other suctioning devices and this probability increases with a longer study duration. For all these reasons, we chose the 72-hour interval for outcome determination.

This outcome is of importance to the families due to the stress and inconvenience of subsequent return visits or hospitalization. It quantifies the morbidity burden of ongoing bronchiolitis symptoms and is also related to financial setback and health care dollars investment. Health care resource utilization represents a powerful marker of bronchiolitis morbidity that has the capacity to alter clinical practice and influence decision makers.

Secondary outcomes:

1- Any medical re-visits for bronchiolitis symptoms to any health care facility within 72 hours of discharge at the index ED visit.

7- Family-initiated bronchiolitis-related medical visits within 72 hours of the index ED visit. We anticipate that patients in the interventional group may have fewer/milder symptoms and less parental anxiety, which may lead to fewer unscheduled visits. In infants discharged from the ED for bronchiolitis, approximately 37% have future family-initiated visits for bronchiolitis during the course of illness which constitute more than a half of all return visits.[50]

8- Emergency Department visits for ongoing/worsening bronchiolitis symptoms within 72 hours of the index ED visit. Patients returning to/referred to the ED represent the most severe end of the disease spectrum and a difference in this outcome will therefore reflect a clinically important difference in symptoms.

9- Normal/near normal feeding at 72 hours. This will be ascertained by the research assistant/research nurse by asking the following question at the e- mail/telephone interview on approximately day 3-4 at the time mutually agreed upon prior to the ED discharge: "Compared to usual, how much food has your infant taken in the last 24 hours? a) normal/near- normal amount [more than about 80% normal]), b) definitely less than normal but more than about 50% normal amount/some difficulty breastfeeding c) very much less than normally (less than 50% normal/major difficulty breastfeeding[16]

10- Normal/near normal sleeping adequacy at 72 hours. This will be ascertained by the research assistant/research nurse by asking the following question at the telephone/e-mail interview on approximately day 3-4: "Based on my child's sleeping pattern, his/her sleep in the past 24 hours was the following compared to usual: a) very much less than normal b) somewhat less than normal c) normally/almost normally), d) somewhat more than normal), e) very much more than normal

11- Parental normal/near normal sleeping adequacy at 72 hours post discharge. This will be based on the question: "In the past 24 hours, how have you (the caregiver) slept compared to usual?" a) very much less than normal b) somewhat less than normal c) normally/almost normally d) somewhat more than normal e) very much more than normal

12- Parental level of satisfaction with their ability to care for their child's illness within 72 hours post discharge from the ED. This will be ascertained by the research assistant/research nurse asking the following question at approximately 72 hours: "Since discharge from the ED, I would rate my level of satisfaction with my ability to care for my child's illness as follows: a) very satisfied, b) satisfied, c) neither satisfied not dissatisfied, d) dissatisfied, e) very dissatisfied." This outcome will reflect the difference in the level of perceived parental ability to provide the needed comfort to their offspring in the two study groups.

Other outcomes: Clinically important side-effects such as prolonged crying with the use of any suctioning device for more than about 5 minutes, epistaxis, vomiting and increased respiratory distress will be tracked. These outcomes are uncommon and the study cannot reasonably be powered for their meaningful analysis. However, these data are important to establish an estimate of a safety profile of the study intervention and of the usual practice.

Outcome Measures Measurement

Baseline (pre-discharge) measurements: The pre-discharge vital signs and saturation will be measured in the ED by the study nurse/research assistants as per usual practice. They will also document the duration of respiratory distress prior to ED arrival, pre-arrival feeding and sleeping status of the patients and review the *ED electronic data records* to confirm subsequent visits for bronchiolitis by 72 hours to our ED. The results of the study outcomes will be

obtained from the caregivers via secure electronic SickKids RED Cap database. For families opting for telephone follow up, trained research assistants will obtain telephone information about outcomes during the standardized *telephone/e- mail follow-ups* at approximately 72 hours. If the families cannot be reached on this day, daily phone calls/e- mails will be made for the next 14 days.

Data Variables Collected

Pre-Screening:

- Site Number
- Registration Date and Time
- Sex
- Age in months
- Inclusion and Exclusion Criteria

Demographics

- Date Enrolled
- Sex
- Age in months

Historical Information

- Details of URI
- Infant feeding and sleeping patterns
- Parental Sleeping patterns
- Details on ED visit
- Familial history of eczema
- Prematurity
- Details on therapy at home

Initial Data

- Triage time
- Vital signs

Medication

- Details on therapy in ED

Discharge Data

- Discharge date and length of stay
- Discharge vital signs

Discharge Therapy

- Details on therapy following ED visit

Follow up

- Adverse event/ Serious Adverse Event
- Details on return visit

Sample Size

The sample size calculation is based on the assessment of the between-group difference in probability of treatment failure. The estimated total re-visit probability in bronchiolitis based on a recently published study was approximately 35% within 72 hours of ED discharge.[49] In our pilot we have found the same rate of treatment failure. This is a superiority study in which the adoption of nasal suctioning will be recommended for future practice if the observed proportion of the primary outcome in this group is significantly lower than in the controls. With 162 patients per arm (324 in total) a two-sided test with a type I error of 0.05 will have 80% power to achieve statistical significance if suctioning reduces the probability of treatment failure from 40% to 25% (i.e. absolute reduction of 15%). This estimate is based on clinically relevant differences agreed upon by study investigators and it also represents an NNT of 7. In the Cochrane review of asthma therapies an NNT of a comparable magnitude led to a change in national practice recommendations.[51] Since bronchiolitis and related medical visits are highly prevalent,[20] this target difference would also have an important economic impact. Based on our previous bronchiolitis trials, the anticipated refusal rate may be 20%. Given the study design and our past experience, the study non-completion rate and loss to follow-up can safely be assumed to be no higher than 5% each. Therefore, to have complete data on 324 patients we plan to randomize 360 (*i.e.* 324/ (1 - 0.05) *(1 – 0.05) and to approach 450 (*i.e.* 360/ (1 - 0.20).

Feasibility

We plan to implement an enrollment schedule similar to the one used in previous similar studies, for a total of 72 hours a week. Extensive weekly coverage is needed since the time of presentation of these children varies. These hours will be covered by several trained on call research nurse/research assistants. Based on our recent bronchiolitis data review from the participating hospitals, we expect 800 (50%) will be screened each season, 480 will be discharged home (40% admission rate), 288 (60%) will meet other eligibility criteria and 230

will be randomized each season (20% refusal rate). Assuming a 5% study non-completion rate and a 5% loss to follow up rate, 206 patients with a full set of data can be expected at all participating EDs each winter. Therefore, 2 bronchiolitis winter seasons represent an adequate timeframe to accrue the needed sample size. Since virtually all bronchiolitis cases occur between December and March, enrollment will be limited to these periods.

Compliance issues

In this study it is important to achieve a balance between adhering to the experimental intervention and to simulate a real-life practice. Since this is a pragmatic trial, no reminders after ED discharge will be implemented. We have also adjusted the sample size to account for the possibility of study non-completion.

We expect that compliance will be enhanced by our provision of the suctioning device and by implementing parental counselling about the association between nasal obstruction and feeding, breathing and sleeping challenges. We will measure compliance during the telephone/e-mail follow up by asking caretakers about the kind of suctioning device used and the frequency of use. We will also conduct an exploratory “as-treated” analysis to determine if those who were compliant with intervention had superior outcomes. The questionnaire also targets the follow up question asking the families if they have used the suctioning device prior to feeds most of the time.

Analyses

The principle of intent-to-treat will be applied to all analyses.

The primary analysis will be a two-sided Fisher’s Exact test to test the null hypothesis that there is no difference in the treatment arms with respect to the probability of treatment failure. This analysis will be performed using a two-sided test of hypothesis with a type I error of 0.05.

Since this trial involves a minimal risk to study subjects, an interim analysis will not be performed.

Secondary analyses:

All secondary analyses will be 2-sided and use a Bonferroni correction (0.05/7=0.007) for the level to declare significance.

7- The Fisher's Exact test will be used to compare the proportions of parents in the two study groups reporting any medical visits for bronchiolitis within approximately 72 hours of discharge from the index ED visit.
8- The Fisher's Exact test will be used to compare the proportions of parents in the two study groups reporting unscheduled (family0initiated) medical visits for bronchiolitis within approximately 72 hours of discharge from the index ED visit.
9- The Fisher's Exact test will be used to compare the proportions of parents reporting ED visits for bronchiolitis within approximately 72 hours of discharge from the index ED visit.
10- The Fisher's Exact test will be used to compare the proportions of parents reporting normal/almost normal feeding pattern at approximately 72 hours.
11- The Fisher's Exact test will be used to compare the proportions of parents reporting normal/almost normal sleeping of their child at approximately 72 hours.
12- The Fisher's Exact test will be used to compare the proportions of parents reporting their own normal/almost normal sleeping at approximately 72 hours.
13- The Fisher's Exact test will be used to compare the proportions of parents reporting their perceived level of satisfaction with care for their child's illness after discharge from the ED as either "very satisfied" or "satisfied" at approximately 72 hours.

As a secondary analysis we will also test for heterogeneity of treatment effect between sites and, if required, apply a random-effects model for comparing treatment groups across sites.

Day to Day Management of the Study

Prior to the study, the co-investigators will educate all ED physicians and nurses/research assistants at the participating sites re the details of the study, hire and train the research staff in the best practice guidelines, accurate data entry and maintaining the study log. The study coordinator will be trained in the various administrative study aspects and is skilled in coordinating multi-site trials. She/he will report to the principal investigator and assist with logistics of the study, organize inter-site communications and check the incoming data for

accuracy and completeness. The co-investigators at all centers will act as a local resource to the clinical research nurse/research assistants and the study managers. We plan several teleconference between collaborators to discuss any outstanding issues. The SickKids study management team will prepare and distribute the study kits to all study centers.

Roles of Applicants:

Dr. Schuh will be responsible for the overall coordination of the study. Drs. Finkelstein, Parkin, Zemek, Plint, Poonai and Eltorki will also be assisting Dr. Schuh. Dr. Coates will also act as respiratory consultant. Dr. Coates has also played a crucial role in piloting the aspiration device.

Trial Steering: Dr Schuh has published numerous single and multi-centre peer-reviewed and competitively funded RCTs of various interventions in asthma, bronchiolitis and croup in the ED. Dr. Schuh will, together with Dr. Plint, a co-investigator and a site PI on the current trial and an experienced CIHR-funded ED scientist, will coordinate the overall study. Dr. Plint has led a multi-site trial of bronchiolitis funded by the CIHR. Dr. Coates has special expertise in nebulization delivery of drugs. Dr Finkelstein has an extensive track record in successful publication of competitive peer-reviewed ED trials; Dr Zemek and Dr Parkin have published numerous pivotal studies enhancing the standard of practice of pediatric acute asthma and Dr Poonai also researches optimal management of common pediatric ailments in the ED.

Safety

Millions of parents worldwide use some form of nasal suctioning in bronchiolitis and this intervention is considered to be safe. Current literature also does not provide evidence of any side effects attributable to the superficial method of suctioning not involving the nasopharynx which will be used in this study. Side effects which may foreseeably happen with this method of suctioning are not likely to be serious and may include irritation of the nasal mucosa, epistaxis, irritability/crying and vomiting. Prolonged crying during suctioning lasting more than about 5 minutes will be tracked in all infants receiving any method of suctioning.

While theoretical side-effects of deep nasal suctioning also include bradycardia, laryngospasm and bronchospasm,[52] we shall not use this invasive sectioning method in this study. There is controversial retrospective inpatient evidence that deep suctioning may result in prolonged length of hospital stay in admitted infants[40,53] due to irritation of the upper airway with resultant edema. In this study we shall utilize a superficial, non-invasive suctioning method not anticipated to result in adverse events.

Adverse Event (AE): An adverse event is any unfavorable or unintended clinical or other occurrence during the study period that may or may not be the result of participation in the research study.

Expected Adverse Events

These include the following as they are part of the natural history of the underlying disease process, such as hospitalization, future health care provider visit, ED return visit, IV rehydration, supplemental oxygen therapy during index or subsequent visits, cough, nasal congestion, respiratory distress, fever, crying post suctioning, irritation to the nasal mucosa, bleeding nose not requiring medical intervention, vomiting. Since expected adverse events are part of the natural history of bronchiolitis, they will not need to be reported as Adverse Events. This information will be recorded in normal study data collection processes.

Serious Adverse Events

Any Serious Adverse Event (SAE) that occurs after suctioning had commenced following ED discharge will be reported to the Research Ethics Board (REB) of all institutions as per local guidelines and to the Principal Investigator within 48 hours and the study subject will be followed until the conclusion of the event.

A SAE in this study will consist of admission to an intensive care unit within 72 hours of discharge from the index ED visit.

Adverse Event Reports

For unexpected adverse events, the site PI will inform their REB as per local guidelines.

Knowledge Translation Strategies

Current evidence-based management of bronchiolitis includes exclusive use of supportive care strategies. Providing the evidence of benefit of the most frequently used such strategy, nasal suctioning, will supply a critical piece of the puzzle in the effective symptomatic management of this common disease with a high nasal obstruction-related morbidity. The results of this trial will help guide optimal bronchiolitis management by health care providers and families and may significantly impact patient-care and healthcare costs.

Future dissemination of the results will include knowledge translation (KT), incorporation of the findings into national and international clinical practice guidelines for management of bronchiolitis as well as into a Cochrane review, presentation at scientific meetings and a peer-reviewed publication. We will also carry out presentations locally, present findings nationally through webinars and meetings of the Canadian Paediatric Society, Canadian Association of Emergency Physicians, and Society of Pediatric Nurses/research assistants and internationally at the Pediatric Academic Societies, American Academy of Pediatrics and the American College of Emergency Physicians.

As active members of national and international expert networks, we have a unique and powerful infrastructure to complete KT of the study results. Integrated KT employing pre-established collaborations with end-knowledge users [Pediatric Emergency Research Canada (PERC), Pediatric Emergency Research Network (PERN), Translating Emergency Knowledge in Kids (TREKK), Canadian Paediatric Society, American Academy of Pediatrics, Canadian Association of Emergency Physicians, American College of Emergency Physicians, Canadian Thoracic Society and American Thoracic Society] will result in broad dissemination to health care providers and the public. Critically, these organizations will incorporate this new evidence into their continuing education aimed at the primary care providers where many families get

counselling on bronchiolitis. Our yearly Pediatric Emergency Conference attended by many primary care providers will further assist in achieving this goal. Using the Twitter, study evidence will also be disseminated to parenting groups, literature on health information for parents. We shall also educate Telehealth where many parents seek health care advice.

We will use PERC, TREKK and PERN to enhance front-line use of our results across Canada and beyond. Specifically, PERC network is part of the world-wide Pediatric Emergency Research Network (PERN), consisting of 122 hospitals on 5 continents. Recognizing that the majority of Canadian children receive emergency care in general EDs, knowledge mobilization is required from the academic pediatric emergency centers to the community. TREKK, funded by the Networks of Centers of Excellence Knowledge Mobilization initiative, aims to accelerate the speed at which the latest knowledge in children's emergency care is put into practice in general EDs. TREKK's knowledge mobilization initiative is grounded in the partnership and exchange between over 30 general EDs across Canada and members of PERC, PERN and KT Canada. Our end-of-grant KT through TREKK will facilitate bridging the research-to-practice gap and raise the overall standard of care for children visiting general EDs with bronchiolitis. Partnership with the Canadian Medical Association and the aforementioned professional organizations ensures that our findings will be employed by health care providers.

We look forward to the time when the emergency physicians and primary care practitioners incorporate suctioning guidelines into discharge instruction materials for their patients with bronchiolitis. We are confident that the proposed study will lead to a higher standard of bronchiolitis care.

References

1. Ralston SL, Lieberthal AS, Meissner HC, et al. Clinical practice guideline: the diagnosis, management, and prevention of bronchiolitis. Pediatrics 2014;134:e1474-502.
2. Shay DK, Holman RC, Newman RD, Liu LL, Stout JW, Anderson LJ. Bronchiolitis-associated hospitalizations among US children, 1980-1996. Jama 1999;282:1440-6.
3. Pelletier AJ, Mansbach JM, Camargo CA, Jr. Direct medical costs of bronchiolitis hospitalizations in the United States. Pediatrics 2006;118:2418-23.
4. Hall CB, Weinberg GA, Iwane MK, et al. The burden of respiratory syncytial virus infection in young children. N Engl J Med 2009;360:588-98.
5. Hasegawa K, Tsugawa Y, Brown DF, Mansbach JM, Camargo CA, Jr. Trends in bronchiolitis hospitalizations in the United States, 2000-2009. Pediatrics 2013;132:28-36.
6. Ehlken B, Ihorst G, Lippert B, et al. Economic impact of community-acquired and nosocomial lower respiratory tract infections in young children in Germany. Eur J Pediatr 2005;164:607-15.
7. Yorita KL, Holman RC, Sejvar JJ, Steiner CA, Schonberger LB. Infectious disease hospitalizations among infants in the United States. Pediatrics 2008;121:244-52.
8. Mansbach JM, Emond JA, Camargo CA, Jr. Bronchiolitis in US emergency departments 1992 to 2000: epidemiology and practice variation. Pediatric Emergency Care 2005;21:242-7.

9. Norwood A, Mansbach JM, Clark S, Waseem M, Camargo CA, Jr. Prospective multicenter study of bronchiolitis: predictors of an unscheduled visit after discharge from the emergency department. Acad Emerg Med 2010;17:376-82.
10. Reynolds EO, Cook CD. The Treatment of Bronchiolitis. J Pediatr 1963;63:1205-7.
11. American Academy of Pediatrics Subcommittee on D, Management of B. Diagnosis and management of bronchiolitis. Pediatrics 2006;118:1774-93.
12. Friedman JN, Rieder MJ, Walton JM, Canadian Paediatric Society ACCDT, Hazardous Substances C. Bronchiolitis: Recommendations for diagnosis, monitoring and management of children one to 24 months of age. Paediatr Child Health 2014;19:485-98.
13. Network SIG. Bronchiolitis in Children: A national clinical guideline2006.
14. Turner T, Wilkinson F, Harris C, Mazza D, Health for Kids Guideline Development G. Evidence based guideline for the management of bronchiolitis. Aust Fam Physician 2008;37:6-13.
15. Barben J, Kuehni CE, Trachsel D, Hammer J, Swiss Paediatric Respiratory Research G. Management of acute bronchiolitis: can evidence based guidelines alter clinical practice? Thorax 2008;63:1103-9.
16. Excellence NIfHaC. Bronchiolitis in children: Diagnosis and management. UK2015.
17. Florin TA, Byczkowski T, Ruddy RM, Zorc JJ, Test M, Shah SS. Variation in the management of infants hospitalized for bronchiolitis persists after the 2006 American Academy of Pediatrics bronchiolitis guidelines. J Pediatr 2014;165:786-92 e1.
18. Christakis DA, Cowan CA, Garrison MM, Molteni R, Marcuse E, Zerr DM. Variation in inpatient diagnostic testing and management of bronchiolitis. Pediatrics 2005;115:878-84.
19. Plint AC, Johnson DW, Wiebe N, et al. Practice variation among pediatric emergency departments in the treatment of bronchiolitis. Acad Emerg Med 2004;11:353-60.
20. Mansbach JM, Emond JA, Camargo CA, Jr. Bronchiolitis in US emergency departments 1992 to 2000: epidemiology and practice variation. Pediatric emergency care 2005;21:242-7.
21. Cheung CR, Smith H, Thurland K, Duncan H, Semple MG. Population variation in admission rates and duration of inpatient stay for bronchiolitis in England. Archives of disease in childhood 2013;98:57-9.
22. Knapp JF, Simon SD, Sharma V. Quality of care for common pediatric respiratory illnesses in United States emergency departments: analysis of 2005 National Hospital Ambulatory Medical Care Survey Data. Pediatrics 2008;122:1165-70.
23. Knapp JF, Hall M, Sharma V. Benchmarks for the emergency department care of children with asthma, bronchiolitis, and croup. Pediatric emergency care 2010;26:364-9.
24. Plint AC, Grenon R, Klassen TP, Johnson DW. Bronchodilator and steroid use for the management of bronchiolitis in Canadian pediatric emergency departments. CJEM 2015;17:46-53.
25. Ralston S, Comick A, Nichols E, Parker D, Lanter P. Effectiveness of quality improvement in hospitalization for bronchiolitis: a systematic review. Pediatrics 2014;134:571-81.
26. Macias CG, Mansbach JM, Fisher ES, et al. Variability in inpatient management of children hospitalized with bronchiolitis. Acad Pediatr 2015;15:69-76.
27. Behrendt CE, Decker MD, Burch DJ, Watson PH. International variation in the management of infants hospitalized with respiratory syncytial virus. International RSV Study Group. Eur J Pediatr 1998;157:215-20.
28. Chirico G, Beccagutti F. Nasal obstruction in neonates and infants. Minerva Pediatr 2010;62:499-505.
29. Walsh S. Infant with feeding difficulties. J Pediatr Health Care 2002;16:204, 9-10.
30. Tamura S, Kurata T. Defense mechanisms against influenza virus infection in the respiratory tract mucosa. Jpn J Infect Dis 2004;57:236-47.

31. Zuercher AW, Coffin SE, Thurnheer MC, Fundova P, Cebra JJ. Nasal-associated lymphoid tissue is a mucosal inductive site for virus-specific humoral and cellular immune responses. J Immunol 2002;168:1796-803.
32. Mallory MD, Shay DK, Garrett J, Bordley WC. Bronchiolitis management preferences and the influence of pulse oximetry and respiratory rate on the decision to admit. Pediatrics 2003;111:e45-51.
33. Baraldi E, Lanari M, Manzoni P, et al. Inter-society consensus document on treatment and prevention of bronchiolitis in newborns and infants. Ital J Pediatr 2014;40:65.
34. Bronchiolitis WGotCPGoA. Clinical Practice Guidelines in the Spanish Healthcare System2010.
35. Verstraete M, Cros P, Gouin M, et al. [Update on the management of acute viral bronchiolitis: proposed guidelines of Grand Ouest University Hospitals]. Arch Pediatr 2014;21:53-62.
36. Oymar K, Skjerven HO, Mikalsen IB. Acute bronchiolitis in infants, a review. Scand J Trauma Resusc Emerg Med 2014;22:23.
37. Nagakumar P, Doull I. Current therapy for bronchiolitis. Arch Dis Child 2012;97:827-30.
38. Moschino L, Mario F, Carraro S, Visentin F, Zanconato S, Baraldi E. Is nasal suctioning warranted before measuring O2 saturation in infants with bronchiolitis? Arch Dis Child 2016;101:114-5.
39. Krugman SD, Bhagtani HR. Parental perception of the effectiveness of treatments for infant nasal congestion. Clin Pediatr (Phila) 2013;52:762-4.
40. Mussman GM, Parker MW, Statile A, Sucharew H, Brady PW. Suctioning and length of stay in infants hospitalized with bronchiolitis. JAMA Pediatr 2013;167:414-21.
41. Zorc JJ, Hall CB. Bronchiolitis: recent evidence on diagnosis and management. Pediatrics 2010;125:342-9.
42. Voets S, van Berlaer G, Hachimi-Idrissi S. Clinical predictors of the severity of bronchiolitis. Eur J Emerg Med 2006;13:134-8.
43. Ducharme FM, Dell SD, Radhakrishnan D, et al. Diagnosis and management of asthma in preschoolers: A Canadian Thoracic Society and Canadian Paediatric Society position paper. Paediatr Child Health 2015;20:353-71.
44. Moher D, Hopewell S, Schulz KF, et al. CONSORT 2010 explanation and elaboration: updated guidelines for reporting parallel group randomised trials. BMJ 2010;340:c869.
45. Lewis JA, Machin D. Intention to treat--who should use ITT? Br J Cancer 1993;68:647-50.
46. Thompson M, Vodicka TA, Blair PS, et al. Duration of symptoms of respiratory tract infections in children: systematic review. BMJ 2013;347:f7027.
47. Schuh S, Freedman S, Coates A, et al. Effect of oximetry on hospitalization in bronchiolitis: a randomized clinical trial. Jama 2014;312:712-8.
48. Pruikkonen H, Uhari M, Dunder T, Pokka T, Renko M. Infants under 6 months with bronchiolitis are most likely to need major medical interventions in the 5 days after onset. Acta Paediatr 2014;103:1089-93.
49. Principi T, Coates AL, Parkin PC, Stephens D, DaSilva Z, Schuh S. Effect of Oxygen Desaturations on Subsequent Medical Visits in Infants Discharged From the Emergency Department With Bronchiolitis. JAMA Pediatr 2016;170:602-8.
50. Petruzella FD, Gorelick MH. Duration of illness in infants with bronchiolitis evaluated in the emergency department. Pediatrics 2010;126:285-90.
51. Rowe BH, Spooner C, Ducharme FM, Bretzlaff JA, Bota GW. Early emergency department treatment of acute asthma with systemic corticosteroids. Cochrane Database Syst Rev 2001:CD002178.
52. Czarnecki ML, Kaucic CL. Infant nasal-pharyngeal suctioning: is it beneficial? Pediatr Nurs 1999;25:193-6, 218.
53. Weisgerber MC, Lye PS, Li SH, et al. Factors predicting prolonged hospital stay for infants with bronchiolitis. J Hosp Med 2011;6:264-70.

Statistical Analysis Plan of SNOT 2018

Sample Size

The sample size calculation is based on the assessment of the between-group difference in probability of treatment failure. The estimated total re-visit probability in bronchiolitis based on a recently published study was approximately 35% within 72 hours of ED discharge.[50] We can therefore reasonably assume this proportion may approach 50% by day 7. Using the 50% estimate is conservative since it requires the largest sample size, providing at least 80% power regardless of the observed proportions. This is a superiority study in which the adoption of nasal suctioning will be recommended for future practice if the observed proportion of the primary outcome in this group is significantly lower than in the controls. With 186 patients per arm (372 in total) a two-sided test with a type I error of 0.05 will have 80% power to achieve statistical significance if suctioning reduces the probability of treatment failure from 50% to 35% (i.e. absolute reduction of 15%). This estimate is based on clinically relevant differences agreed upon by study investigators and it also represents an NNT of 7. In the Cochrane review of asthma therapies an NNT of a comparable magnitude led to a change in national practice recommendations.[52] Since bronchiolitis and related medical visits are highly prevalent,[20] this target difference would also have an important economic impact. Based on our previous bronchiolitis trials, the anticipated refusal rate may be 20%. Given the study design and our past experience, the study non-completion rate and loss to follow-up can safely be assumed to be no higher than 5% each. Therefore, to have complete data on 372 patients we plan to randomize 412 (*i.e.* 372/ (1 - 0.05) *(1 – 0.05) and to approach 515 (*i.e.* 412/(1 - 0.20).

Analyses

The principle of intent-to-treat will be applied to all analyses. Per-protocol analyses will also be conducted.

Primary Outcome:

The primary analysis will be a two-sided Fisher's Exact test to test the null hypothesis that there is no difference in the treatment arms with respect to the probability of treatment failure. This analysis will be performed using a two-sided test of hypothesis with a type I error of 0.05. Since this trial involves a minimal risk to study subjects, an interim analysis will not be performed.

Analyses of the Secondary Outcomes:

All secondary analyses will be 2-sided and use a Bonferroni correction (0.05/6=0.0083) for the level to declare significance.

14- The Fisher's Exact test will be used to compare the proportions of parents in the two study groups reporting all medical visits for bronchiolitis within 7 days of discharge from the index ED visit.

15- The Fisher's Exact test will be used to compare the proportions of parents reporting ED visits for bronchiolitis within 7 days of discharge from the index ED visit.

16- The Fisher's Exact test will be used to compare the proportions of parents reporting normal/almost normal feeding pattern on day 7.

17- The Fisher's Exact test will be used to compare the proportions of parents reporting normal/almost normal sleeping of their child on day 7.

18- The Fisher's Exact test will be used to compare the proportions of parents reporting their own normal/almost normal sleeping on day 7.

19- The Fisher's Exact test will be used to compare the proportions of parents reporting their perceived level of satisfaction with care for their child's illness after discharge from the ED as either "very satisfied" or "satisfied" on day 7.

As a secondary analysis we will also test for heterogeneity of treatment effect between sites and, if required, apply a random-effects model for comparing treatment groups across sites.

Specifically, we shall perform adjusted analyses using generalized linear mixed modelling to control for randomization stratification by age group and site, with the site treated as a random effect.

Statistical Analysis Plan SNOT 2019

Sample Size

The sample size calculation is based on the assessment of the between-group difference in probability of treatment failure. The estimated total re-visit probability in bronchiolitis based on a recently published study was approximately 35% within 72 hours of ED discharge.[49] In our pilot we have found the same rate of treatment failure. This is a superiority study in which the adoption of nasal suctioning will be recommended for future practice if the observed proportion of the primary outcome in this group is significantly lower than in the controls. With 162 patients per arm (324 in total) a two-sided test with a type I error of 0.05 will have 80% power to achieve statistical significance if suctioning reduces the probability of treatment failure from 40% to 25% (i.e. absolute reduction of 15%). This estimate is based on clinically relevant differences agreed upon by study investigators and it also represents an NNT of 7. In the Cochrane review of asthma therapies an NNT of a comparable magnitude led to a change in national practice recommendations.[51] Since bronchiolitis and related medical visits are highly prevalent,[20] this target difference would also have an important economic impact. Based on our previous bronchiolitis trials, the anticipated refusal rate may be 20%. Given the study design and our past experience, the study non-completion rate and loss to follow-up can safely be assumed to be no higher than 5% each. Therefore, to have complete data on 324 patients we plan to randomize 360 (*i.e.* 324/ (1 - 0.05) *(1 – 0.05) and to approach 450 (*i.e.* 360/ (1 - 0.20).

Analyses

The principle of intent-to-treat will be applied to all analyses. The estimated effect will be quantified as a relative risk difference with the corresponding 95% confidence intervals (CI) and p-values. We shall also use the Fisher-exact test to examine each of the two primary outcome components.

<u>Primary Outcome:</u>

The primary analysis will be a two-sided Fisher's Exact test to test the null hypothesis that there is no difference in the treatment arms with respect to the probability of treatment failure. This analysis will be performed using a two-sided test of hypothesis with a type I error of 0.05. Since this trial involves a minimal risk to study subjects, an interim analysis will not be performed.

<u>Analyses of the Secondary Outcomes:</u>

All secondary analyses will be 2-sided and use a Bonferroni correction (0.05/6=0.008) for the level to declare significance.

20- The Fisher's Exact test will be used to compare the proportions of parents in the two study groups reporting any medical visits for bronchiolitis within approximately 72 hours of discharge from the index ED visit.

21- The Fisher's Exact test will be used to compare the proportions of parents reporting ED visits for bronchiolitis within 72 hours of discharge from the index ED visit.

22- The Fisher's Exact test will be used to compare the proportions of parents reporting normal/almost normal feeding pattern at 72 hours.

23- The Fisher's Exact test will be used to compare the proportions of parents reporting normal/almost normal sleeping of their child at 72 hours.

24- The Fisher's Exact test will be used to compare the proportions of parents reporting their own normal/almost normal sleeping at 72 hours.

25- The Fisher's Exact test will be used to compare the proportions of parents reporting their perceived level of satisfaction with care for their child's illness after discharge from the ED as either "very satisfied" or "satisfied" at 72 hours.

As a secondary analysis we will also test for heterogeneity of treatment effect between sites and, if required, apply a random-effects model for comparing treatment groups across sites. Specifically, we shall perform adjusted analyses using generalized linear mixed modelling to control for randomization stratification by age group and site, with the site treated as a random effect.
