# Supplementary material for: Nasal Suctioning Therapy Among Infants With Bronchiolitis Discharged Home From the Emergency Department: A Randomized Clinical Trial
Source: JAMA Netw Open. 2023 Oct 19;6(10):e2337810. doi: 10.1001/jamanetworkopen.2023.37810 (PMC10587796; doi:10.1001/jamanetworkopen.2023.37810)
Supplement: Supplement 2. — eAppendix 1. Follow-Up Questionnaire eAppendix 2. Feeding and Sleeping Adequacy Questions eAppendix 3. Satisfaction With Care After Discharge eTable 1. Secondary Outcomes of the Trial: Per-Protocol Analyses eTable 2. Changes in Feeding and Sleeping During the Trial: Per-Protocol Analyses [file jamanetwopen-e2337810-s002.pdf]

Supplemental Online Content

Schuh S, Coates AL, Sweeney J, et al; Pediatric Emergency Research Canada (PERC) Network. Nasal suctioning therapy among infants with bronchiolitis discharged home from the emergency department: a randomized clinical trial. *JAMA Netw Open*. 2023;6(10):e2337810. doi:10.1001/jamanetworkopen.2023.37810

eAppendix 1. Follow-Up Questionnaire

eAppendix 2. Feeding and Sleeping Adequacy Questions

eAppendix 3. Satisfaction With Care After Discharge

eTable 1. Secondary Outcomes of the Trial: Per-Protocol Analyses

eTable 2. Changes in Feeding and Sleeping During the Trial: Per-Protocol Analyses

This supplemental material has been provided by the authors to give readers additional information about their work.

© 2023 Schuh S et al. *JAMA Network Open.*

eAppendix 1: FOLLOW-UP QUESTIONNAIRE

A. RETURN VISIT

1) Since going home from the Emergency Department (when you/your child started participating in this research study), has your child visited a doctor for bronchiolitis?

Yes [ ] No [ ]

If NO, please continue to page 2 (Section B. FEEDING)

If YES, how many times has your child visited a doctor for bronchiolitis?

(choose only one)

☐ One ☐ Two ☐ Three

For each visit to a doctor for bronchiolitis, please answer the following questions:

	Visit 1	Visit 2	Visit 3
Visit 1: Did the Emergency Department doctor recommend this visit?<br><br>Visit 2-3: Was the visit recommended by your doctor?	[ ] Yes<br>[ ] No	[ ] Yes<br>[ ] No	[ ] Yes<br>[ ] No
Did this visit to the doctor happen because you were concerned about your child's bronchiolitis?	[ ] Yes<br>[ ] No	[ ] Yes<br>[ ] No	[ ] Yes<br>[ ] No
Where did you go?<br>a. Family doctor/pediatrician<br>b. Emergency Department<br>c. Walk in Clinic<br>d. Other, specify below	[ ] a<br>[ ] b<br>[ ] c<br>[ ] d	[ ] a<br>[ ] b<br>[ ] c<br>[ ] d	[ ] a<br>[ ] b<br>[ ] c<br>[ ] d
If Other (d), specify			

© 2023 Schuh S et al. *JAMA Network Open.*

2) Since going home from our Emergency Department, has your child been admitted to a hospital (to a hospital ward) for bronchiolitis? Yes [ ] No [ ]

B. FEEDING

Compared to usual, how has your child fed in the past 24 hours?

(Mark your answer by putting X on one of the circles)

Normal/almost normal feeding (more than about 80% normal bottle intake or no/minimal difficulty breastfeeding)	Below normal but more than ½ usual bottle intake or difficulty breastfeeding	Very much less than normal (less than ½ usual bottle intake or major difficulty breastfeeding)
●	●	●

C. SLEEPING

CHILD

(Mark your answer by putting X on one of the circles)

Compared to usual, how has your **CHILD** slept in the past 24 hours?

Very much less than normal	Less than normal	Normal/ almost normal	Somewhat more than normal	Very much more than normal
●	●	●	●	●

PARENT or MAIN CAREGIVER (the person who is caring for the child overnight)

Compared to usual, how have **YOU/PARENT or the MAIN CAREGIVER** slept in the past 24 hours?

Very much less than normal	Less than normal	Normal/ almost normal	Somewhat more than normal	Very much more than normal
●	●	●	●	●

D. ABILITY TO CARE FOR YOUR CHILD'S ILLNESS

(Mark your answer by putting X on one of the circles)

© 2023 Schuh S et al. *JAMA Network Open.*

How would you or the main caregiver rate your level of satisfaction with your ability to care for your child while he/she has been sick (since the initial Emergency Department visit 3 days ago)?

Very satisfied	Satisfied	Neither satisfied nor dissatisfied	Dissatisfied	Very Dissatisfied
☺	☺	😐	😕	☹
●	●	●	●	●

E. NOSE SUCTION

1) Since going home from the Emergency Department, did you use any of the suctioning device(s) below to clean your child's nose? *(check **all** that apply):*

[ ] Zo-Li battery-operated nasal aspirator

[ ] Bulb nasal aspirator

[ ] Other battery-operated nasal aspirator (specify): ______________________

For example:

[ ] Mouth to nose human suction nasal aspirator

For example: Hydrasense, Naspira, Nosefrida, or any other similar device.

2) How often did you use any of the following suctioning devices in the last 3 days?

Name of Suctioning Device	Never	Rarely	Occasionally	Often (before most feeds)
Zo-Li nasal aspirator	●	●	●	●
Bulb nasal aspirator	●	●	●	●
Other battery-operated nasal aspirator	●	●	●	●

© 2023 Schuh S et al. *JAMA Network Open.*

Mouth to nose human suction nasal aspirator e.g. Hydrasense, Naspira, Nosefrida	●	●	●	●

3) Since going home from the Emergency Department, how many days have you used this suctioning device?

*Check **all** that apply*:

Name of Suctioning Device	Never	Less than 1 day	1-2 days	More than 2 days
Zo-Li nasal aspirator	●	●	●	●
Bulb nasal aspirator	●	●	●	●
Other battery-operated nasal aspirator	●	●	●	●
Mouth to nose human suction nasal aspirator e.g. Hydrasense, Naspira, Nosefrida	●	●	●	●
	Please continue to question number 4	*Please continue to question number 4*	*Please continue to question number 4*	*Please skip question number 4 and go to question number 5*

4) If you used the suctioning rarely, never or stopped suctioning before 3 days, what were the main reasons?

*Check **all** that apply*:

Name of Suctioning Device	My child got better and did not need suctioning	Suctioning device does not help my child feed or breathe better	Suctioning device was making my child uncomfortable	Suctioning device was not suctioning properly	Other *(Please specify below)*
Zo-Li nasal aspirator	●	●	●	●	●
Bulb nasal aspirator	●	●	●	●	●
Other battery-operated nasal aspirator	●	●	●	●	●
Mouth to nose human suction nasal aspirator e.g. Hydrasense, Naspira, Nosefrida	●	●	●	●	●

© 2023 Schuh S et al. *JAMA Network Open.*

If Other, please specify:

____

____

5) Did any of the following occur immediately after the use of this device? *Check all that apply*:

	Prolonged screaming more than 5 minutes	Blood dripping from the nose (active nosebleed)	Marked worsening of breathing problem	Vomiting	Nothing to report	Other *(Please specify below)*
Zo-Li nasal aspirator	●	●	●	●	●	●
Bulb nasal aspirator	●	●	●	●	●	●
Other battery-operated nasal aspirator	●	●	●	●	●	●
Mouth to nose human suction nasal aspirator e.g. Hydrasense, Naspira, Nosefrida	●	●	●	●	●	●

If you have nothing to report please go to question number 8.

If Other, please specify:

____

____

6) If any of these problems occurred, did you see a doctor to get these problems treated?

Yes [ ] No [ ]

If NO, please continue to question number 8

7) If YES, what problems made you see the doctor? *Check **all** that apply*:

	Prolonged screaming	Blood dripping from the nose	Marked worsening of	Vomiting	Other

© 2023 Schuh S et al. *JAMA Network Open.*

	more than 5 minutes	(active nosebleed)	breathing problem		*(Please specify below)*
Zo-Li nasal aspirator					
Bulb nasal aspirator					
Other battery-operated nasal aspirator					
Mouth to nose human suction nasal aspirator e.g. Hydrasense, Naspira, Nosefrida					

If Other, please specify:

____

____

8) How satisfied were you with the device you have used in the past 3 days?

(Mark your answer by putting X on one of the circles)

	Very satisfied	Satisfied	Neither satisfied Nor dissatisfied	Dissatisfied	Very Dissatisfied
Zo-Li nasal aspirator					
Bulb nasal aspirator					
Other battery-operated nasal aspirator					
Mouth to nose human suction nasal aspirator e.g. Hydrasense, Naspira, Nosefrida					
	Please skip question 9 and continue to question 10	*Please skip question 9 and continue to question 10*	*Please skip question 9 and continue to question 10*	*Please continue to question number 9*	*Please continue to question number 9*

9) If you are dissatisfied or very dissatisfied, what are the reasons? *Check all that apply*:

	Noise from the suctioning device	Device was difficult/awkward to use	Excessive irritability of my baby	Poor/insufficient suctioning	Suctioning seemed too strong	Other (Please specify below)
Zo-Li nasal aspirator						

© 2023 Schuh S et al. *JAMA Network Open.*

Bulb nasal aspirator						
Other battery-operated nasal aspirator						
Mouth to nose human suction nasal aspirator e.g. Hydrasense, Naspira, Nosefrida						

If Other, please specify:

____

____

10) Will you use this same nose suctioning device again in the future?

	Very much agree	Agree	Neither agree nor disagree	Disagree	Very much disagree
Zo-Li nasal aspirator					
Bulb nasal aspirator					
Other battery-operated nasal aspirator					
Mouth to nose human suction nasal aspirator e.g. Hydrasense, Naspira, Nosefrida					

11) Did you use nose drops/spray with suctioning? Yes [ ] No [ ]

(For example: Salinex that helps to clear and clean blocked nose passages)

Thank you very much for participating in this very important study and completing this follow-up questionnaire!

© 2023 Schuh S et al. *JAMA Network Open.*

eAppendix 2: Feeding and Sleeping Adequacy Questions

Prior to randomization and at 72 hours, the parents were asked about their infant's feeding and sleeping adequacy and their own sleeping adequacy over the past 24 hours by asking the following Likert scale questions:

Feeding:

"In the past 24 hours, how has your child fed compared to usual?"

a) normal/almost normal feeding amount (more than about 80% normal bottle intake or no/minimal difficulty breastfeeding), b) below normal feeding but more than ½ usual bottle intake or some difficulty breastfeeding c) feeding very much less than normal (less than ½ usual bottle intake or major difficulty breastfeeding);

Sleeping:

"In the past 24 hours, how has your child slept compared to usual?"

a) very much less than normal, b) less than normal), c) normal/almost normal, d) somewhat more than normal, e) very much more than normal.

Parental Sleep:

"In the past 24 hours, how have you (the caregiver) slept compared to usual?"

a) very much less than normal, b) somewhat less than normal, c) normal/almost normal, d) somewhat more than normal, e) very much more than normal.

© 2023 Schuh S et al. *JAMA Network Open.*

eAppendix 3: Satisfaction With Care After Discharge

The caregivers' satisfaction with their ability to manage their child's illness was measured by their response to the following question: "How would you rate your level of satisfaction with your ability to care for your child while he/she has been sick (since the initial ED visit 3 days ago)?

a) very satisfied, b) satisfied, c) neither satisfied nor dissatisfied, d) dissatisfied, e) very dissatisfied.

© 2023 Schuh S et al. *JAMA Network Open.*

eTable 1. Secondary Outcomes of the Trial: Per-Protocol Analyses

Outcome	**Minimal suction N=76**	**Enhanced Suction N=106**	**Unadjusted difference (95% CI)**	**p-value**	**Adjusted difference[a] (95% CI)**	**p-value**
**All medical re-visits[c]**	17 (22.4)	22 (20.8)	0.02(-0.11 to 0.14)	0.86	0.02(-0.09 to 0.13)	0.71
**ED re-visits**	10 (13.2)	13 (12.3)	0.01(-0.09 to 0.11)	1.00	0.01 (-0.07to 0.09)	0.80[b]
**Normal feeding at 72 hours[d]**	54 (71.1)	74 (69.8)	0.01(-0.12 to 0.15)	0.87	-0.00 (-0.14 to 0.13)	0.95
**Normal sleep at 72 hours**	40 (52.6)	49 (46.2)	0.06(-0.08 to 0.21)	0.45	0.06 (-0.08 to 0.21)	0.39
**Normal parental sleep**	27 (35.5)	45 (42.5)	-0.07 (-0.21 to 0.07)	0.36	0.07 (-0.07 to 0.22)	0.30
**Satisfied with care after ED discharge[e]**	59 (77.6)	94 (88.7)	-0.11 (-0.00 to 0.21)	0.06	0.10 (-0.01 to 0.21)	0.07

[a] Post hoc adjustment for stratification at randomization for age group, with site as a random effect.

[b] Based on logistic regression analysis

[c] Defined as any unscheduled visit for bronchiolitis to any medical facility or provider within 72 hours of ED discharge

[d] Defined as >80% normal fluid intake

[e] Defined as satisfied or very satisfied with bronchiolitis care after discharge

© 2023 Schuh S et al. *JAMA Network Open.*

eTable 2. Changes in Feeding and Sleeping During the Trial: Per-Protocol Analyses

Outcome	Minimal Suction N=76			Enhanced Suction N=106			Unadjusted		Adjusted	
	Baseline N (%)	72 hr N (%)	Unadjusted difference (95% CI)	Baseline N (%))	72hr N (%)	Unadjusted difference (95% CI)	Difference-in-differences[a] (95% CI)	P	Difference-in-differences (95% CI)	P
**Normal feeding[b]**	28 (36.8)	54 (71.1)	0.34 (0.19 to 0.49)	34 (32.1)	74 (69.8)	0.38 (0.25 to 0.50)	0.04 (-0.16 to 0.23)	0.72	0.03 (-0.16 to 0.23)	0.73
**Normal sleeping**	15 (19.7)	40 (52.6)	0.33 (0.18 to 0.47)	21 (19.8)	49 (46.2)	0.26 (0.14 to 0.39)	0.06 (-0.25 to 0.12)	0.50	0.06 (-0.25 to 0.12)	0.51
**Normal parental sleep**	12 (15.8)	27 (35.5)	0.20 (0.06 to 0.33)	16 (15.1)	45 (42.5)	0.27 (0.16 to 0.39)	0.08 (-0.10 to 0.26)	0.40	0.08 (-0.10 to 0.25)	0.41

[a] Post hoc adjustment for stratification at randomization for age group, with site as a random effect.

[b] Defined as >80% normal fluid intake

© 2023 Schuh S et al. *JAMA Network Open.*
