# Supplementary material for: Nasal Suctioning Therapy Among Infants With Bronchiolitis Discharged Home From the Emergency Department: A Randomized Clinical Trial
Source: JAMA Netw Open. 2023 Oct 19;6(10):e2337810. doi: 10.1001/jamanetworkopen.2023.37810 (PMC10587796; doi:10.1001/jamanetworkopen.2023.37810)
Supplement: Supplement 3. — Pediatric Emergency Research Canada (PERC) Network Members [file jamanetwopen-e2337810-s003.pdf]

Supplemental Online Content: Nonauthor Collaborators
*First name, last name, and suffix (if applicable) are required and will appear in PubMed.

*Group Name(s): Pediatric Emergency Research Canada (PERC) Network							
***First Name and Middle Initial(s)**	***Last Name**	***Suffix (eg, Jr, III)**	Academic Degrees	Institution	Location (city, state/province, country)	Role or Contribution, eg, chair, principal investigator	Group (if more than 1 Group listed in the byline) and/or Subgroup (eg, Steering Committee)
Redjana	Carciumaru		MD, MSc	McMaster Children's Hospital	Hamilton, ON, Canada	study coordinator	PERC
Kamary	CoriolanoDaSilva		PhD	Schulich School of Medicine and Den	London, ON, Canada	study coordinator	PERC
Kristina	Vogel		MSc	Children's Hospital of Eastern Ontario	Ottawa, ON, Canada	study coordinator	PERC
Tanveer	Colins		MSc	Hospital for Sick Children	Toronto, ON, Canada	database manager	
