# Supplementary material for: Nasal Suctioning Therapy Among Infants With Bronchiolitis Discharged Home From the Emergency Department: A Randomized Clinical Trial
Source: JAMA Netw Open. 2023 Oct 19;6(10):e2337810. doi: 10.1001/jamanetworkopen.2023.37810 (PMC10587796; doi:10.1001/jamanetworkopen.2023.37810)
Supplement: Supplement 4. — Data Sharing Statement [file jamanetwopen-e2337810-s004.pdf]

Data Sharing Statement

Schuh. Nasal Suctioning Therapy Among Infants With Bronchiolitis Discharged Home From the Emergency Department. *JAMA Netw Open*. Published October 13, 2023.
doi:10.1001/jamanetworkopen.2023.37810

Data
Data available: Yes
Data types: Deidentified participant data
How to access data: rahim.moinedin@utoronto.ca
When available: With publication

Supporting Documents
Document types: Statistical/analytic code
How to access documents: rahim.moinedin@utoronto.ca
When available: With publication

Additional Information
Who can access the data: Researchers whose proposed used of data has been approved for a specific study
Types of analyses: For a pre-specified and approved purpose
Mechanisms of data availability: After approval of a specific proposal and with a signed data access agreement
